# *In Vitro* Disease Modeling of Hermansky-Pudlak Syndrome Type 2 Using Human Induced Pluripotent Stem Cell-Derived Alveolar Organoids

**DOI:** 10.1016/j.stemcr.2019.01.014

**Published:** 2019-02-14

**Authors:** Yohei Korogi, Shimpei Gotoh, Satoshi Ikeo, Yuki Yamamoto, Naoyuki Sone, Koji Tamai, Satoshi Konishi, Tadao Nagasaki, Hisako Matsumoto, Isao Ito, Toyofumi F. Chen-Yoshikawa, Hiroshi Date, Masatoshi Hagiwara, Isao Asaka, Akitsu Hotta, Michiaki Mishima, Toyohiro Hirai

**Affiliations:** 1 Department of Respiratory Medicine, Graduate School of Medicine, Kyoto University, Kyoto 606-8507, Japan; 2 Department of Drug Discovery for Lung Diseases, Graduate School of Medicine, Kyoto University, Kyoto 606-8501, Japan; 3 Department of Thoracic Surgery, Graduate School of Medicine, Kyoto University, Kyoto 606-8507, Japan; 4 Department of Anatomy and Developmental Biology, Graduate School of Medicine, Kyoto University, Kyoto 606-8501, Japan; 5 Department of Fundamental Cell Technology, Center for iPS Cell Research and Application, Kyoto University, Kyoto 606-8507, Japan; 6 Department of Clinical Application, Center for iPS Cell Research and Application, Kyoto University, Kyoto 606-8507, Japan

**Keywords:** iPSC, Hermansky-Pudlak syndrome, HPS, lamellar body, alveolar type 2 cell, pulmonary surfactant, pluripotent stem cell, pulmonary fibrosis, alveolar organoid, MX35

## Abstract

It has been challenging to generate *in vitro* models of alveolar lung diseases, as the stable culture of alveolar type 2 (AT2) cells has been difficult. Methods of generating and expanding AT2 cells derived from induced pluripotent stem cells (iPSCs) have been established and are expected to be applicable to disease modeling. Hermansky-Pudlak syndrome (HPS) is an autosomal recessive disorder characterized by dysfunction of lysosome-related organelles, such as lamellar bodies (LBs), in AT2 cells. From an HPS type 2 (HPS2) patient, we established disease-specific iPSCs (HPS2-iPSCs) and their gene-corrected counterparts. By live cell imaging, the LB dynamics were visualized and altered distribution, enlargement, and impaired secretion of LBs were demonstrated in HPS2-iPSC-derived AT2 cells. These findings provide insight into the AT2 dysfunction in HPS patients and support the potential use of human iPSC-derived AT2 cells for future research on alveolar lung diseases.

## Highlights

• HPS2-iPSCs and cHPS2-iPSCs were generated from HPS2 patient fibroblasts
• Anti-NaPi2b antibody was useful for isolating AT2 cells from human lung and AOs
• The enlargement and abnormal distribution of LBs were observed in HPS2-AOs
• Impaired surfactant secretion was demonstrated in HPS2-AOs

## Graphical Abstract

**Figure:**
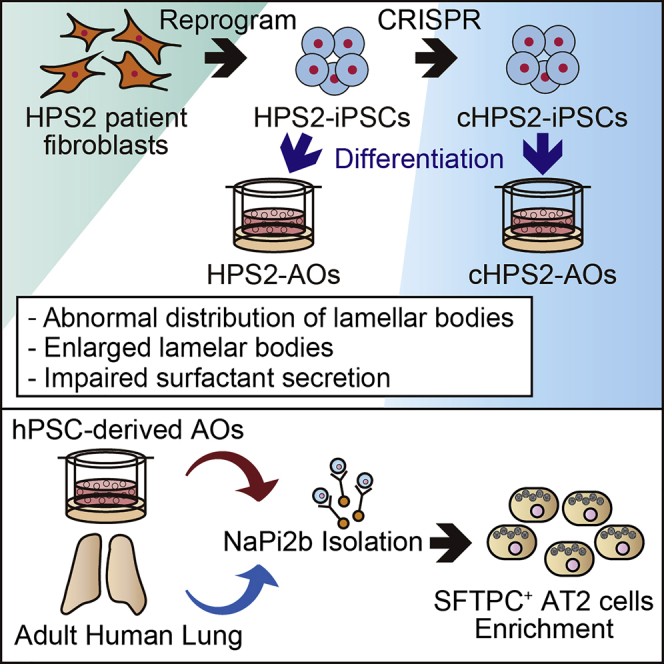

## Teaser

Pulmonary surfactant secretion by alveolar type 2 (AT2) cells is crucial for alveolar homeostasis. Gotoh and colleagues generated Hermansky-Pudlak syndrome type 2 (HPS2) patient-derived iPSCs and their gene-corrected counterparts and differentiated them into alveolar organoids (AOs). In HPS2-AOs, aberrant lamellar bodies and impaired surfactant secretion were demonstrated. Human iPSC-derived AOs might be useful for investigating alveolar lung diseases.

## Introduction

Alveolar type 2 (AT2) cells are tissue stem cells that maintain homeostasis of the alveolar region of the lung (Barkauskas et al., 2013). They secrete pulmonary surfactant to prevent alveolar collapse and contribute to the host defense of the lung (Whitsett et al., 2015). Lamellar bodies (LBs), characteristic organelles of mature AT2 cells, are lysosome-related organelles (LROs) involved in the storage and secretion of pulmonary surfactant and are often affected in alveolar lung diseases, including hereditary pulmonary fibrosis (PF) (Nakatani et al., 2000, Whitsett et al., 2015). However, the mechanism underlying the LB degeneration and AT2 cell dysfunction in human alveolar lung diseases is not well understood due to poor accessibility and the difficulty of isolating and culturing primary AT2 cells. Induced pluripotent stem cells (iPSCs) are expected to overcome these limitations. We previously established methods for generating human pluripotent stem cell (hPSC)-derived alveolar and airway cells in organoids (Gotoh et al., 2014, Konishi et al., 2016) and successfully expanded hPSC-derived AT2 cells in alveolar organoids (AOs) (Yamamoto et al., 2017).

Hermansky-Pudlak syndrome (HPS) is a rare autosomal recessive hereditary disease caused by mutations in the genes involved in the formation and maturation of LROs and characterized by oculocutaneous albinism and bleeding diathesis (El-Chemaly and Young, 2016). Among the ten subtypes, patients with HPS1, HPS2, and HPS4 can suffer from PF. HPS is a disease of LROs, and the abnormal enlargement of LBs in AT2 cells was reported in both HPS patients (Nakatani et al., 2000) and mouse models (Lyerla et al., 2003). HPS2 is an extremely rare subtype of HPS that is caused by mutations of the *AP3B1* gene, which encodes the β3A subunit of the AP-3 complex, which is involved in intracellular membrane traffic. It was previously reported that approximately 40% of HPS2 patients had PF and that 78% of HPS2 patients with PF were children (Jessen et al., 2013).

In this study, we generated HPS2 patient-derived iPSCs (HPS2-iPSCs) and gene-corrected iPSCs (cHPS2-iPSCs) and differentiated them into AOs (HPS2-AOs and cHPS2-AOs, respectively). Based on the comparison of these AOs, we report the AT2 cell dysfunction of HPS2-AOs.

## Results

### Generation of HPS2-iPSCs and cHPS2-iPSCs

**Figure 1:**
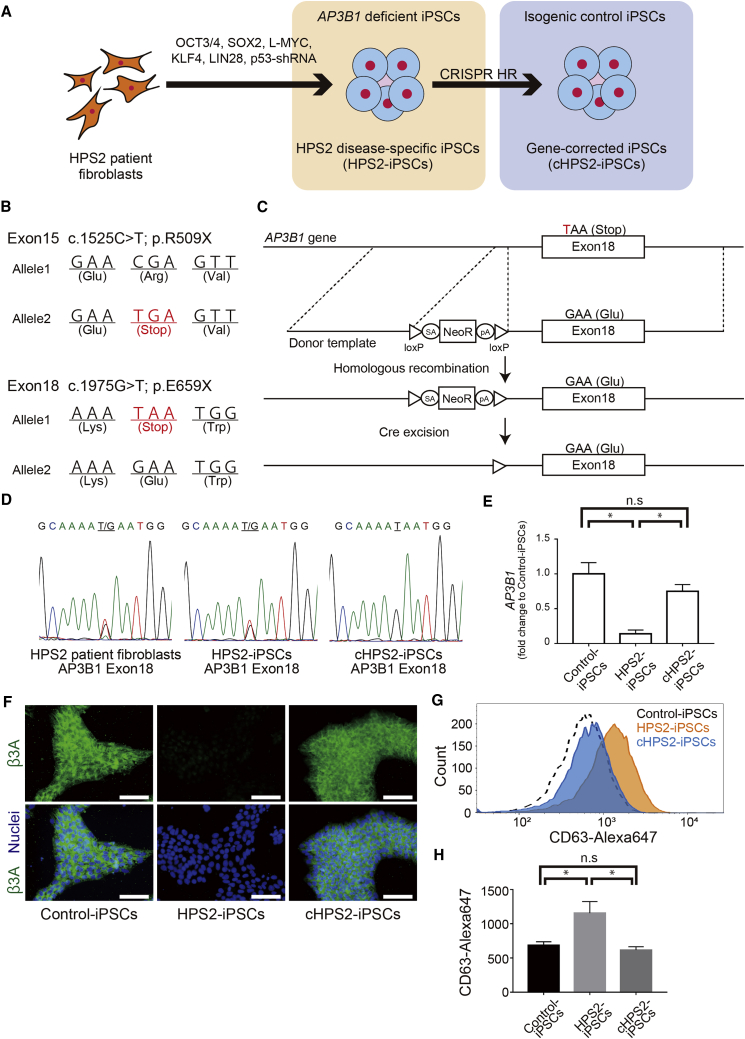
Generation of HPS2-iPSCs and cHPS2-iPSCs (A) Schematic overview of the generation of HPS2-iPSCs and cHPS2-iPSCs. (B) Different mutations in each allele of the patient fibroblasts. (C) Strategy for correcting the mutation in exon 18. (D) Sequence data of exon 18 in donor fibroblasts, HPS2-iPSCs, and cHPS2-iPSCs. The mutation was corrected in cHPS2-iPSCs. (E) qRT-PCR of *AP3B1* in each cell line. 201B7 was used for control iPSCs (mean ± SEM, n = 3 independent experiments). A one-way ANOVA with Tukey's multiple comparisons test was used. ^∗^p < 0.05; n.s., not significant. (F) IF staining of the β3A subunit of AP-3 complex in each iPSC line. 201B7 was used for control iPSCs. Scale bars, 100 μm. (G) Surface CD63 expression in control iPSCs, HPS2-iPSCs, and cHPS2-iPSCs. 201B7 was used for control iPSCs. (H) Median fluorescence intensity of CD63-Alexa647 (mean ± SEM, n = 3 independent experiments). A one-way ANOVA with Tukey's multiple comparisons test was used. ^∗^p < 0.05; n.s., not significant. See also Figure S1.

HPS2-iPSCs were established from patient fibroblasts obtained from the Coriell Institute for Medical Research (GM17890) (Figure 1A). The HPS2 patient donor had compound heterozygous nonsense mutations in exon 15 and 18 of the *AP3B1* gene and he was histologically diagnosed with nonspecific interstitial pneumonitis at 20 months of age (Huizing et al., 2002) (Figure 1B). Next, cHPS2-iPSCs were generated from HPS2-iPSCs by using CRISPR/Cas9-mediated homologous recombination (Li et al., 2015) (Figure 1C). We targeted the mutation on exon 18, because it was not possible to design a single guide RNA to hybridize with the mutation on exon 15. After G418 selection and limiting dilution, 36 out of 132 clones (27%) had the donor template at the target locus. After Cre excision, we chose a res69-5 clone for the subsequent experiments. The sequencing data showed that the mutation in exon 18 was corrected in cHPS2-iPSCs (Figures 1D and S1A). There were no indels at 58 predicted off-target sites (Table S1). The *AP3B1* transcript level was decreased to 14% ± 5% in HPS2-iPSCs and restored to 75% ± 10% in cHPS2-iPSCs, in comparison with normal control iPSCs (Figure 1E), which was indicative of nonsense-mediated mRNA decay (NMD) in HPS2-iPSCs, as reported in donor cells (Huizing et al., 2002). In immunofluorescence (IF) staining, the β3A subunit was almost absent in HPS2-iPSCs and was restored in cHPS2-iPSCs (Figure 1F). Western blotting demonstrated the absence of AP3B1 and the decrease of AP3M1 in HPS2-iPSCs, consistent with the previous report by Kook et al. (2018) (Figure S1B). Both HPS2-iPSCs and cHPS2-iPSCs expressed undifferentiated markers and showed no abnormal karyotypes (Figures S1C and S1D). The pluripotency was demonstrated by the teratoma formation (Figure S1E) and there was no integration of reprogramming vectors in genomic DNA (Figure S1F). CD63 molecules interact with AP-3 complex via its tyrosine-based targeting motif and are sorted to lysosomes (Rous et al., 2002). Since CD63 is mis-sorted to the cell surface in AP-3 dysfunction, the function of AP-3 complex is assayable by flow cytometry of CD63 (Dell'Angelica et al., 1999). In HPS2-iPSCs, the increased cell surface CD63 expression was observed in comparison with control iPSCs and cHPS2-iPSCs, suggesting the dysfunction of AP-3 complex in HPS2-iPSCs and its restoration in cHPS2-iPSCs (Figures 1G and 1H).

### Comparison of the Methods of NKX2-1^+^ Cell Isolation

**Figure 2:**
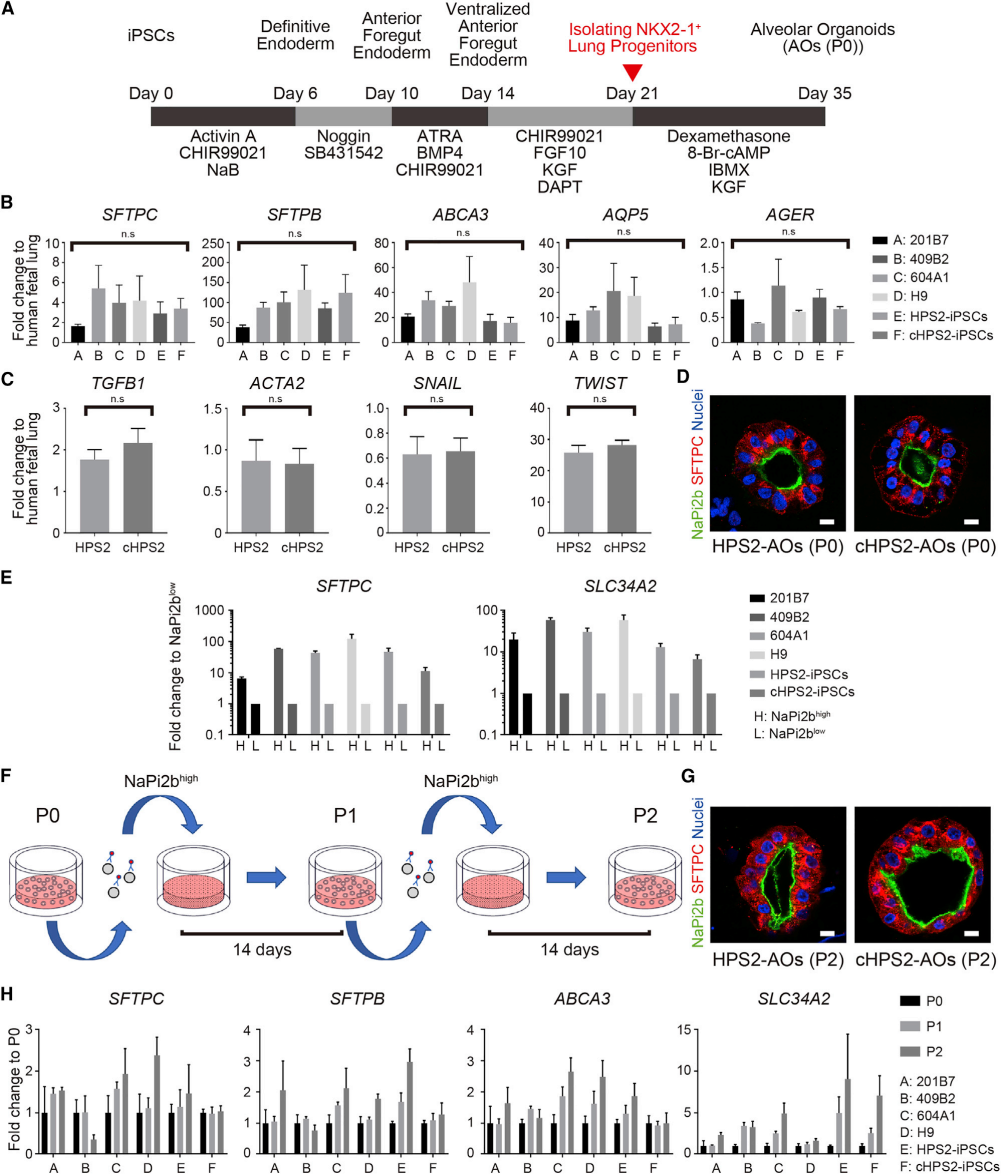
Generation of iPSC-derived AOs and Their Expansion Based on the Expression of NaPi2b (A) Schematic overview of the generation of hPSC-derived AOs. (B) qRT-PCR of AT1 and AT2 markers in AOs (P0) (mean ± SEM, n = 3 independent experiments). Kruskal-Wallis with Dunn's multiple comparisons test was used. n.s., not significant. (C) qRT-PCR of fibrosis-related markers in AOs (P0) (mean ± SEM, n = 5 independent experiments). The Mann-Whitney test was used. n.s., not significant. (D) Confocal IF staining of AOs (P0) derived from HPS2-iPSCs and cHPS2-iPSCs. Scale bars, 10 μm. (E) qRT-PCR of *SFTPC* and *SLC34A2* in NaPi2b^high^ and NaPi2b^low^ cells sorted from AOs (P0) (mean ± SEM, n = 3 independent experiments). (F) Schematic overview of the subculture of NaPi2b^high^ cells in AOs. NaPi2b^high^ cells were passaged every 2 weeks. (G) Confocal IF staining of AOs (P2) subcultured using anti-NaPi2b antibodies. Scale bars, 10 μm. (H) qRT-PCR of AT2 markers in subcultured AOs (P0–P2) (mean ± SEM, n = 3 independent experiments). See also Figures S2 and S3.

The isolation of NKX2-1^+^ lung progenitor cells is a critical step in the generation of lung epithelial cells from hPSCs. We compared the isolation efficiency of previously reported sorting methods, carboxypeptidase M (CPM) and CD47 combined with or without CD26 (Figure S2A) (Gotoh et al., 2014, Hawkins et al., 2017). At day 21 of our induction protocol (Figure 2A), CPM^high^ cells contained the most NKX2-1^+^ cells in all hPSC lines (Figures S2B and S2C). Since there was no interaction between the cell lines and sorting methods analyzed by two-way ANOVA, the samples from different cell lines were all analyzed together. As a result, CPM-based sorting was able to isolate more NKX2-1^+^ cells than CD47-based methods (Figure S2D).

### Induction of AOs from HPS2-iPSCs and cHPS2-iPSCs

Upon CPM-based sorting, NKX2-1^+^ lung progenitor cells derived from both HPS2-iPSCs and cHPS2-iPSCs formed AOs after three-dimensional co-culture with human fetal lung fibroblasts (HFLFs). The expression levels of representative AT2 markers (*SFTPC*, *SFTPB*, and *ABCA3*) and alveolar type 1 (AT1) markers (*AQP5* and *AGER*) were not significantly different among the AOs derived from control hPSCs and HPS2-iPSCs and cHPS2-iPSCs (Figure 2B). The fibrosis-related markers (*TGFB1*, *ACTA2*, *SNAIL*, and *TWIST*) showed no differences between HPS2-AOs and cHPS2-AOs (Figure 2C), consistent with the findings of the animal study in that the *Ap3b1* mutant (Pearl) mice did not develop PF spontaneously (Young et al., 2007).

### Utility of NaPi2b as a Surface Antigen for the Subculture of AOs

NaPi2b encoded by *SLC34A2* is a multi-transmembrane protein expressed on AT2 cells, and MX35 is a monoclonal antibody that recognizes its extracellular domain (Yin et al., 2008). IF staining showed that SFTPC^+^ cells in both adult and fetal human lung tissues expressed NaPi2b (Figure S2E). We investigated whether this antibody was useful for the isolation of AT2 cells from the adult human lung. The proportion of SFTPC^+^ AT2 cells was 84.2% ± 6.5% in the NaPi2b^high^ population and 84.8% ± 3.6% in the HT2-280^high^ population (Figures S2F–S2I). qRT-PCR showed that the AT2 marker expression was concentrated in the NaPi2b^high^ population, whereas AT1 and airway markers were enriched in the NaPi2b^low^ population, suggesting that AT2 cells were selectively isolated in the NaPi2b^high^ population (Figures S2J).

NaPi2b was also expressed on the luminal surface of SFTPC^+^ cells in hPSC-derived AOs regardless of the *AP3B1* genotype (Figure 2D). The NaPi2b^high^ population contained more SFTPC^+^ cells than the NaPi2b^low^ population (Figures S3A–S3C), which was supported by the different levels of *SFTPC* and *SLC34A2* in both populations (Figure 2E). When NaPi2b^high^ cells were co-cultured with HFLFs, AT2 cells were successfully subcultured in AOs, which also expressed AT2 cell markers with a stepwise increase of *SLC34A2* (Figures 2F–2H). In P2-AOs, NaPi2b was maintained on the luminal surface of SFTPC^+^ cells in both HPS2-AOs and cHPS2-AOs, similarly to P0-AOs (Figure 2G). We also investigated HT2-280 for isolating AT2 cells in AOs, but the number of HT2-280^+^ cells was too small for subculture or analysis (Figures S3D and S3E). These findings indicated that anti-NaPi2b monoclonal antibody (MX35) was more useful than HT2-280 for subculturing AT2 cells in hPSC-derived AOs.

### Abnormal Distribution and Aberrant Structures of LBs in HPS2-AOs

**Figure 3:**
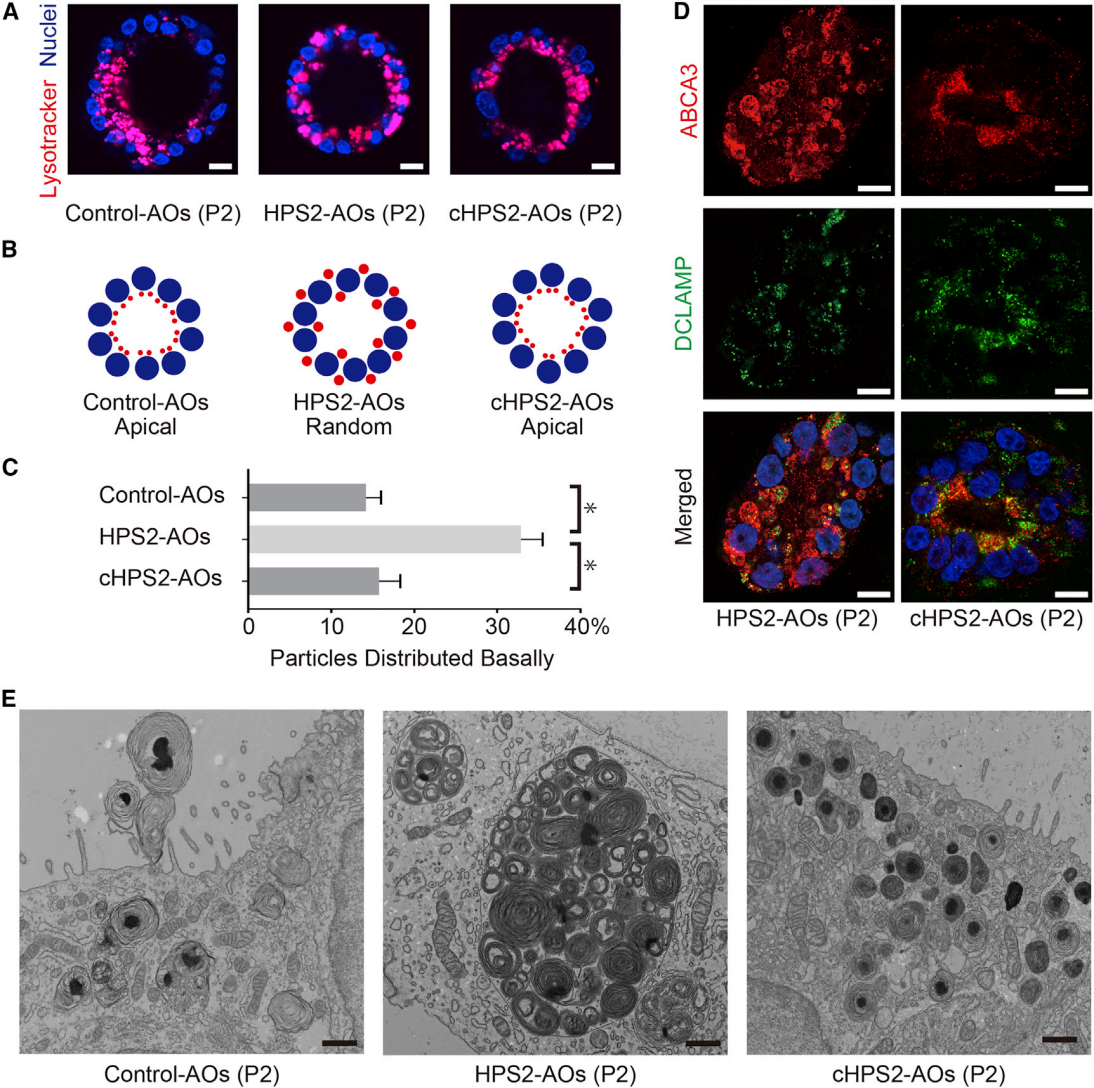
Morphological Features of HPS2-AOs and cHPS2-AOs (A) Live cell imaging of AOs (P2) derived from control iPSCs, HPS2-iPSCs, and cHPS2-iPSCs, respectively. Scale bars, 10 μm. (B) Schematic illustration of the pattern of LT^+^ organelle distribution in AOs. (C) Quantitative analysis of LT^+^ organelles distributed basally in the spheroids (mean ± SEM, n = 22–32 from three independent experiments). Kruskal-Wallis with Dunn's multiple comparisons test was used. ^∗^p < 0.05. (D) Confocal IF staining of ABCA3 and DCLAMP in HPS2-AOs and cHPS2-AOs (P2). Scale bars, 10 μm. (E) Electron micrographs of AOs (P2) from each cell line. Scale bars, 1 μm. See also Figure S3.

LBs in AOs were examined via live cell imaging using LysoTracker (LT), a fluorescent probe used for visualizing LBs (Haller et al., 1998, Yamamoto et al., 2017). Interestingly, whereas the LT^+^ organelles were gathered on the apical side of the constituent epithelial cells in control AOs and cHPS2-AOs, they were distributed randomly in HPS2-AOs (Figures 3A–3C). This distributional difference was more clearly observed in P2-AOs than in P0-AOs (Figures 3A and S3F). In addition, the LT^+^ organelles in HPS2-AOs seemed enlarged in P2-AOs (Figures 3A and 3B). In IF staining, ABCA3, a limiting membrane protein of the LBs (Yamano et al., 2001), was located on apically distributed vesicles in DCLAMP^+^ cells of cHPS2-AOs whereas it was located on the enlarged and randomly distributed vesicles in those of HPS2-AOs, suggesting that LT^+^ organelles corresponded to LBs (Figure 3D). In electron microscopy, giant LBs (>5 μm in diameter) were occasionally observed in HPS2-AOs, whereas the LBs in control AOs or cHPS2-AOs were of a normal size (1–2 μm in diameter) (Figure 3E). Furthermore, abnormally formed lysosome-like organelles were frequently observed in HPS2-AOs (Figure S3G), consistent with a previous report on Pearl mice (Zhen et al., 1999). We therefore concluded that altered distribution and enlargement of LBs with abnormally formed organelles were the phenotype of HPS2-AOs.

### Impaired Surfactant Secretion from HPS2-AO-Derived Cells

**Figure 4:**
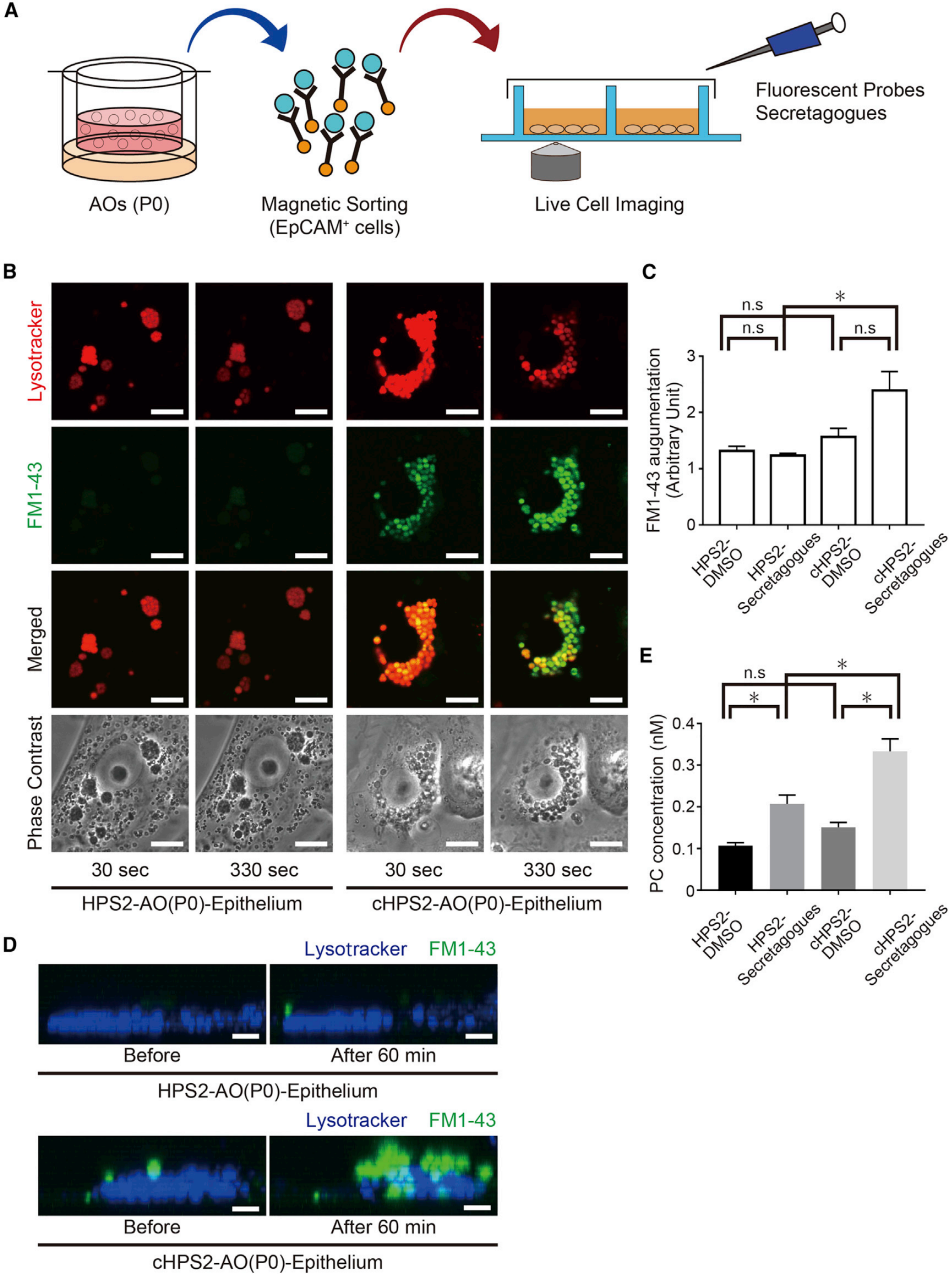
LB Secretion Assay for AT2 Cells Derived from HPS2-iPSCs and cHPS2-iPSCs (A) Schematic overview of the LB secretion assay. (B) Confocal images of HPS2-AO(P0) and cHPS2-AO(P0) epithelial cells at 30 and 330 s after stimulation with secretagogs. Phase-contrast images show the location of LBs and nuclei. Scale bars, 10 μm. (C) Quantitative comparison of the FM1-43 augmentation levels (mean ± SEM, n = 7–10 from three independent experiments). Kruskal-Wallis with Dunn's multiple comparisons test was used. ^∗^p < 0.05; n.s., not significant. (D) z-stack images of LT and FM1-43 staining before and after stimulation with secretagogs. Scale bars, 5 μm. (E) Phosphatidylcholine (PC) concentration of the supernatant of secretagog-stimulated epithelial cells from AOs (mean ± SEM, n = 11 from 11 independent experiments). Kruskal-Wallis with Dunn's multiple comparisons test was used. ^∗^p < 0.05; n.s., not significant. See also Figure S3 and Video S1.

As decreased secretion of pulmonary surfactant was reported in HPS1 and HPS2 double-mutant mice (Pale Ear/Pearl) (Guttentag et al., 2005), we evaluated the pulmonary surfactant secretion of HPS2-AOs and cHPS2-AOs. EpCAM^+^ cells of both AOs were reseeded on coverglass chambers, and live cell imaging was performed after staining with LT and FM1-43, a fluorescent probe that labels free lipids and which has been used to evaluate the exocytosis of LBs from primary AT2 cells (Haller et al., 1998) (Figure 4A). After stimulation with a secretagog cocktail, the fluorescence intensity of FM1-43 was increased in cHPS2-AO epithelial cells but not in HPS2-AO epithelial cells (Figures 4B and 4C; Video S1). Consistently, z-stack images suggested that more FM1-43 stained vesicles were secreted from cHPS2-AO cells than from HPS2-AO cells (Figure 4D). Furthermore, the concentration of phosphatidylcholine in culture supernatant was significantly reduced in HPS2-AO-derived cells (Figure 4E). These findings suggested that HPS2-AO cells were less prone to secrete the surfactant, consistent with the findings that Pearl mice showed decreased secretion of kidney lysosomal enzymes (Novak and Swank, 1979). In addition, there was no significant change in the levels of FM1-43 augmentation between the enlarged and normal LBs in HPS2-AOs (Figures 4B, S3H, and S3I), suggesting that impaired LB secretion in HPS2-AO cells was caused by dysfunction of the AP-3 complex rather than the size of the LBs.

Video S1. Time-Lapse Video of the LB Secretion Assay The LBs stained with LT (red) turned yellow-green due to the fluorescence of FM1-43 (green) in the cHPS2-AO(P0) epithelium, whereas the color of LBs showed no change in the HPS2-AO(P0) epithelium. Scale bars, 5 μm.

## Discussion

We established the HPS2-iPSCs from patient fibroblasts and their gene-corrected cHPS2-iPSCs. Although the mutation in exon 15 persisted in cHPS2-iPSCs, we consider the results obtained from the experiments using cHPS2-iPSCs to be reliable for three reasons. First, the increased CD63 in HPS2-iPSCs was restored in cHPS2-iPSCs, suggesting that AP-3 complex works sufficiently, even in the presence of the exon 15 mutation. Second, HPS2 is an autosomal recessive disease and it was reported that the mother of the donor, who had the nonsense mutation in exon 15 but not in exon 18, had no symptoms of HPS2 (Huizing et al., 2002). Finally, the amount of *AP3B1* mRNA is very low in HPS2-iPSCs due to NMD, and the aberrant protein production is negligibly low. For these reasons, we consider cHPS2-iPSCs to be an appropriate control for HPS2-iPSCs.

The mechanism underlying the formation of giant LBs in HPS is unknown. Since there was a report suggesting that enlarged lysosomes have impaired motility, even in wild-type cells (Perou and Kaplan, 1993), we investigated whether the size of LBs affected the FM1-43 augmentation levels in the HPS2-AO epithelium. Our results suggested that the impaired LB secretion in HPS2-AO cells was caused by the dysfunction of AP-3 complex rather than the size of the LBs, which was consistent with the idea that AP-3 is required for the sorting of proteins involved in the movement of LROs along microtubules (Clark et al., 2003). We considered that the enlargement of the LBs in HPS2-AOs is a result of impaired surfactant secretion rather than its cause. In addition, the abnormal distribution of LBs seen in HPS2-AOs was analogous to the previous report on abnormalities in cytotoxic T lymphocytes (CTLs) in HPS2 patients, in which lytic granules, the LROs of CTLs, failed to move to the secretory surface of the cells (Clark et al., 2003).

Pulmonary surfactant plays critical roles in the maintenance of the alveolar environment and its deficiency causes respiratory distress syndrome (RDS) in newborns. In addition, mutations in genes associated with LBs, such as *ABCA3* and *SFTPB*, are typically lethal in neonates because of defective surfactant metabolism (Whitsett et al., 2015). In this study, the stimulated secretion of pulmonary surfactant was decreased in HPS2-AOs, but it was reported that HPS2 patients do not present RDS and that Pearl mice do not develop RDS or PF spontaneously in their lifetimes (Young et al., 2007). Our finding that basal secretion was preserved, not abrogated, in HPS2-AOs might explain why HPS2 patients and Pearl mice do not present RDS. Further studies are needed to investigate this discrepancy. It also remains to be elucidated whether decreased surfactant secretion may play some roles in the pathogenesis of PF in HPS2 patients, although decreased surfactant secretion has been reported in PF patients (Schmidt et al., 2002).

In this study, we validated three methods related to lung stem cell research. First, for the isolation NKX2-1^+^ lung progenitor cells, CPM-based and CD47-based methods were compared (Gotoh et al., 2014, Hawkins et al., 2017). The CPM^high^ population contained more NKX2-1^+^ cells than the CD47^high^CD26^low^ population, although our induction protocol had originally been developed to fit CPM-based sorting. The second involved the isolation of AT2 cells from the adult human lung by the anti-NaPi2b antibody. Currently, HT2-280 is used for isolating AT2 cells from adult lung (Gonzalez et al., 2010). We demonstrated that the proportions of SFTPC^+^ AT2 cells in the NaPi2b^high^ and the HT2-280^high^ populations were equivalent and that other AT2 cell markers were enriched in the NaPi2b^high^ population. Finally, we demonstrated that the anti-NaPi2b antibody was also useful for passaging hPSC-derived AOs without establishing reporter cell lines. As we previously reported, the *SFTPC* expression in hPSC-AOs decreased without passaging, and it was necessary to isolate SFTPC^+^ cells and reseed them into Matrigel with HFLFs in order to maintain the AT2 marker expression (Yamamoto et al., 2017). Although this is a limitation of the current hPSC-AO system, progress has been made in maintaining more AT2 markers in organoids in comparison with conventional two-dimensional culture. In this study, P2-AOs were used to analyze the distribution or morphological characteristics of LBs because P2-AOs contained more LBs than P0-AOs (Figure 3). On the other hand, to analyze the LB secretion (Figure 4), EpCAM^+^ cells, not NaPi2b^high^ cells, from P0-AOs were used to obtain sufficient cells.

It is necessary for disease modeling to recapitulate the functions of the cells responsible for the disease. While hPSC-derived lung organoids including both airway and alveolar lineages have been reported (Chen et al., 2017), there are few reports of organoids focusing on alveolar cells and their functions (Jacob et al., 2017, Yamamoto et al., 2017). To our knowledge, no reports have verified the secretion of pulmonary surfactant in both hPSC-derived AOs and in HPS patient-derived cells. Human iPSC-derived AT2 cells might overcome the limited availability and aid in discovering therapeutic agents via disease modeling in a dish.

## Experimental Procedures

### Live Cell Imaging

After AOs or reseeded cells were stained with fluorescent probes, all of the samples were examined under an FV10i-LIV confocal microscope (Olympus) with a 60× objective under 5% CO_2_ at 37°C. For the LB secretion assay, the medium was supplemented with a secretagog cocktail so that the final concentration of each component was 5 μM forskolin, 15 μM ATP, 150 nM ionomycin, and 150 nM phorbol 12-myristate 13-acetate. For further details, see Supplemental Experimental Procedures.

### Ethics

The use of H9 hESCs was approved by the Ministry of Education, Culture, Sports, Science and Technology (MEXT), Japan. The animal experiments were approved by the Animal Research Committee of Kyoto University. The use of human lung samples was approved by the Ethics Committee of Kyoto University Graduate School and Faculty of Medicine.

### Statistical Analyses

All error bars indicate the SEM. Quantified data represent the findings of three or more independent experiments. The statistical tests used are shown in each legend. All statistical analyses were performed using the Prism7 software program (GraphPad).

## Author Contributions

Y.K. and S.G. conceived and designed the study. Y.K., S.G., S.I., Y.Y., N.S., K.T., S.K., and T.N. performed the experiments. Y.K., S.G., I.A., and A.H. generated HPS2-iPSCs and cHPS2-iPSCs. Y.K., S.G., S.I., Y.Y., T.F.C.-Y., and H.D. contributed to AT2 cell isolation from human lung tissues. Y.K., S.G., S.I., and Y.Y. analyzed the data. Y.K. and S.G. wrote the manuscript through fruitful discussions with and supervision by H.M., I.I., M.H., M.M., and T.H.

## Supplemental Information

Document S1. Supplemental Experimental Procedures, Figures S1–S3, and Tables S1–S4

Document S2. Article plus Supplemental Information
