# Supplementary material for: In Vitro Disease Modeling of Hermansky-Pudlak Syndrome Type 2 Using Human Induced Pluripotent Stem Cell-Derived Alveolar Organoids
Source: Stem Cell Reports. 2019 Feb 14;12(3):431–40. doi: 10.1016/j.stemcr.2019.01.014 (PMC6409438; doi:10.1016/j.stemcr.2019.01.014)
Supplement: Document S1. Supplemental Experimental Procedures, Figures S1–S3, and Tables S1–S4 [file mmc1.pdf]

Stem Cell Reports, Volume *12*

Supplemental Information

In Vitro Disease Modeling of Hermansky-Pudlak Syndrome Type 2 Using Human Induced Pluripotent Stem Cell-Derived Alveolar Organoids

Yohei Korogi, Shimpei Gotoh, Satoshi Ikeo, Yuki Yamamoto, Naoyuki Sone, Koji Tamai, Satoshi Konishi, Tadao Nagasaki, Hisako Matsumoto, Isao Ito, Toyofumi F. Chen-Yoshikawa, Hiroshi Date, Masatoshi Hagiwara, Isao Asaka, Akitsu Hotta, Michiaki Mishima, and Toyohiro Hirai

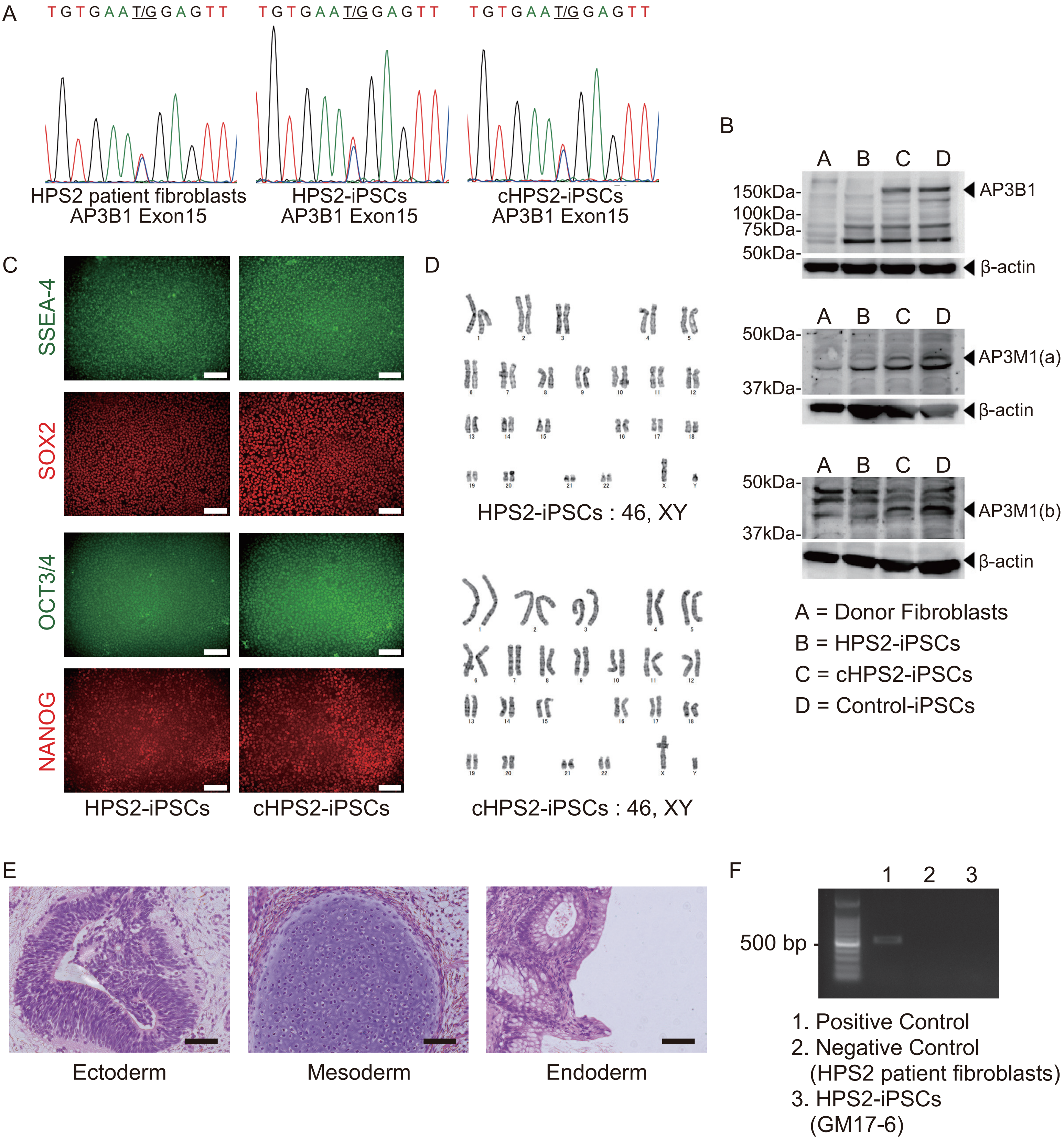

Figure S1. Generation of HPS2-iPSCs and cHPS2-iPSCs, Related to Figure 1.
(A) Sequence data of exon 15 in donor fibroblasts, HPS2-iPSCs and cHPS2-iPSCs. The mutation in exon 15 was not corrected in cHPS2-iPSCs. (B) Western blotting analyses of AP3B1 and AP3M1. AP3M1 was analyzed with two primary antibodies ((a) NBP1-76589 and (b) NBP2-27068). 201B7 iPSCs were used for control iPSCs. (C) IF staining of undifferentiated markers SSEA-4, SOX2, OCT3/4 and NANOG in HPS2-iPSCs and cHPS2-iPSCs. Scale bars, 100 μm. (D) The G-banding analysis of the karyotypes of HPS2-iPSCs and cHPS2-iPSCs. (E) Hematoxylin-eosin staining of teratoma derived from HPS2-iPSCs in immunodeficient mice. The three panels show ectoderm (neuronal cells), mesoderm (cartilage), and endoderm (gland) tissues. Scale bars, 100 μm. (F) The PCR to detect the integration of reprogramming vectors in genomic DNA. PCR products were not detected in HPS2-iPSCs.

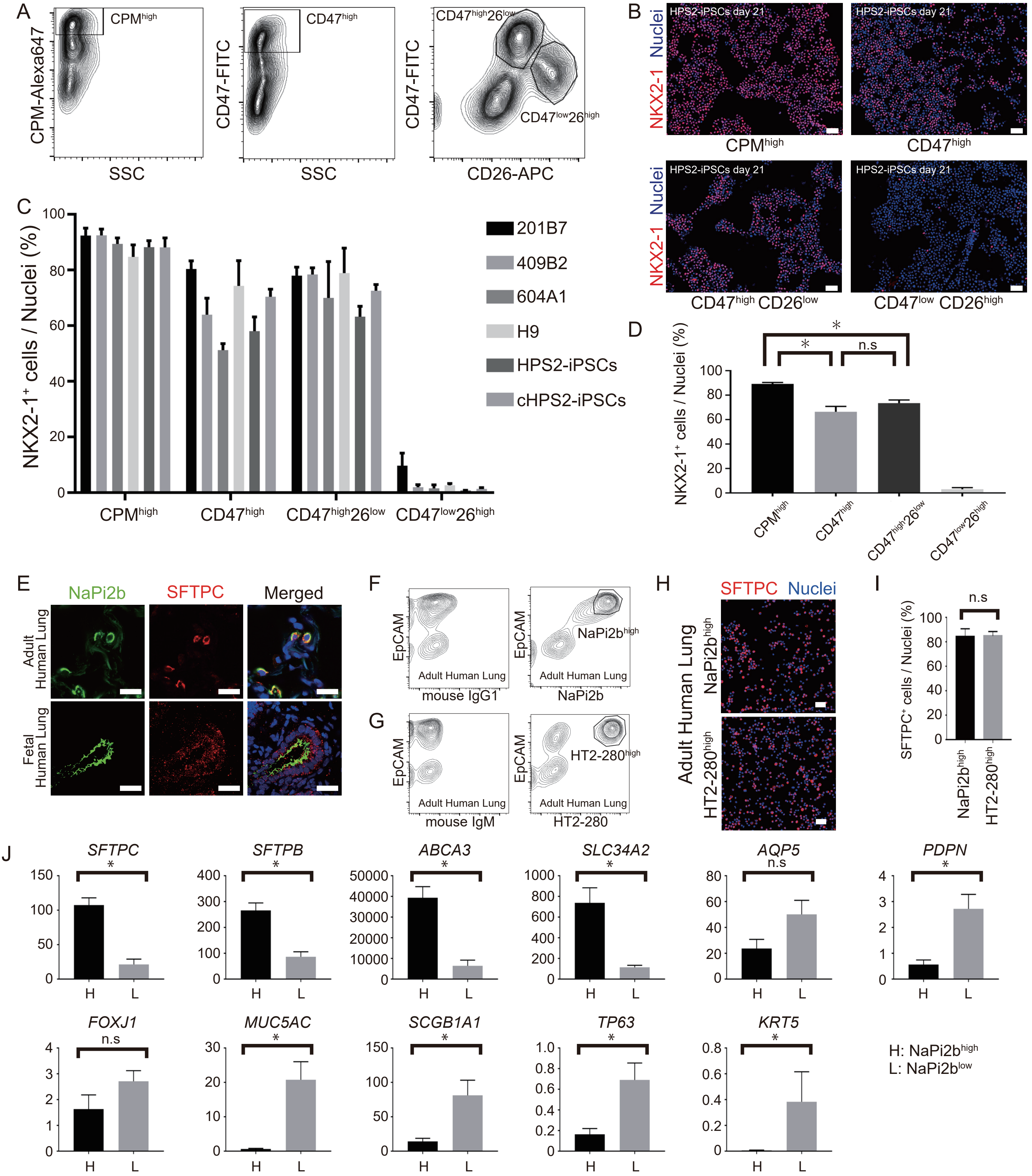

Figure S2. Validation of Methods of Isolating NKX2-1⁺ Cells and Passaging of AT2 Cells Based on the NaPi2b Expression, Related to Figure 2.

(A) Flow cytometric analyses of iPSC-derived lung progenitor cells on day 21. The gating of CPM^{high}, $CD47^{high}$, $CD47^{high}CD26^{low}$ and $CD47^{low}CD26^{high}$ are shown. (B) IF staining of cytospin samples of each cell population with anti-NKX2-1 antibody. Scale bars, 50μm. (C, D) The quantitative comparison of the proportion of $NKX2\text{-}1^{+}$ cells (mean ± S.E.M., n = 3 for (C) and n = 18 for (D), 3 independent experiments). A two-way ANOVA with Tukey' s multiple comparisons test was used. *$p < 0.05$. n.s., not significant. (E) Confocal IF staining of adult and fetal human lung tissues with anti-NaPi2b and anti-SFTPC antibodies. Scale bars, 25 μm. (F, G) Flow cytometric analyses of adult human lung. CD45-negative cells are plotted and the gating of $NaPi2b^{high}$ and $HT2\text{-}280^{high}$ is shown. (H) IF staining of cytospin samples isolated from adult human lung. Scale bars, 50 μm. (I) The proportion of $SFTPC^{+}$ cells in $NaPi2b^{high}$ and $HT2\text{-}280^{high}$ cell population (mean ± S.E.M., n = 3 donors). The Mann-Whitney test was used. n.s., not significant. (J) The transcript levels of lineage-specific markers of the $NaPi2b^{high}$ and $NaPi2b^{low}$ population from adult human lung (mean ± S.E.M., n = 4 donors). The Mann-Whitney test was used. *$p < 0.05$. n.s., not significant.

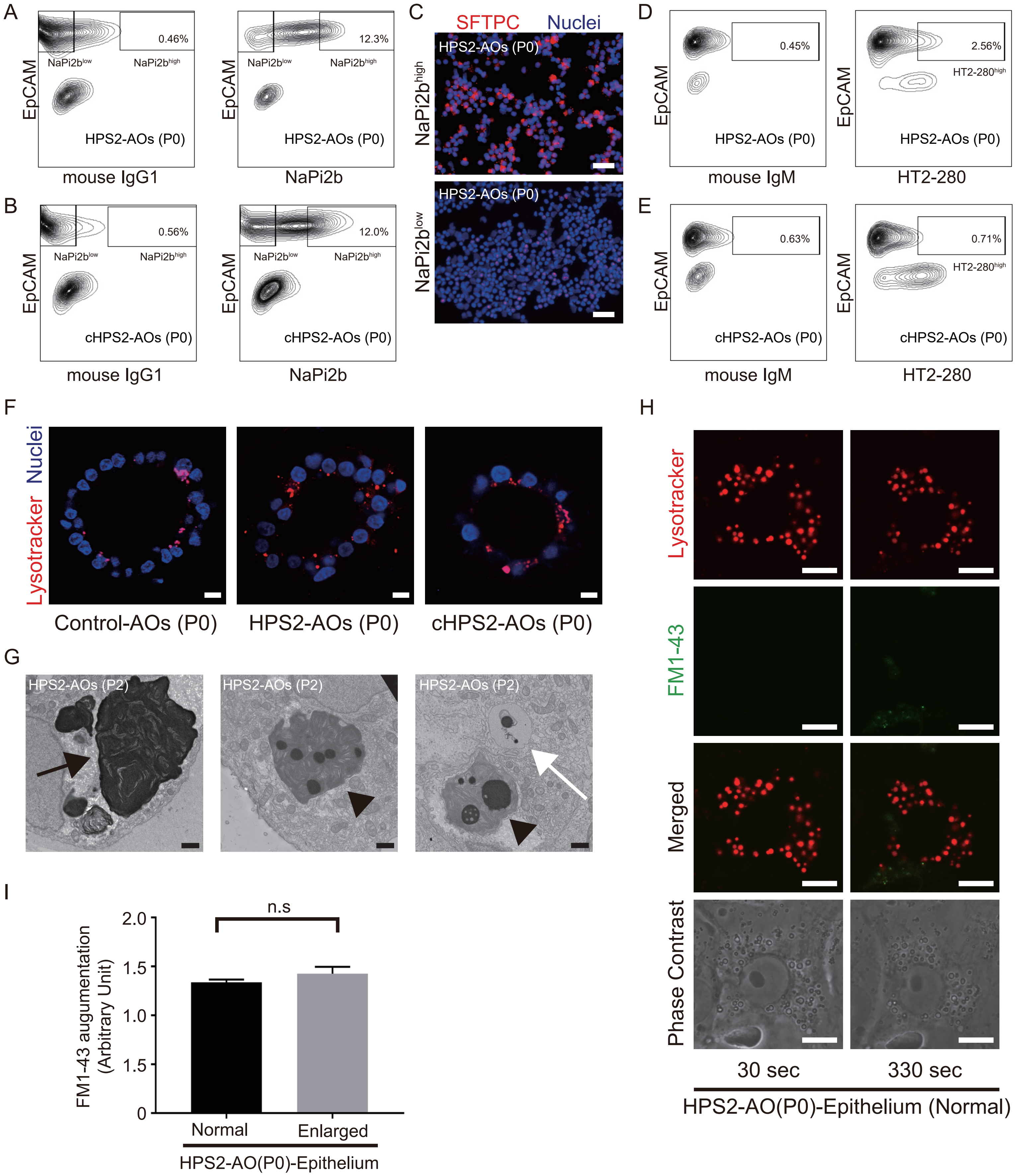

A
EpCAM
NaPi2blow
NaPi2bhigh
0.46%
HPS2-AOs (P0)
mouse IgG1
12.3%
NaPi2b
B
0.56%
cHPS2-AOs (P0)
12.0%
C
SFTPC
Nuclei
NaPi2bhigh
NaPi2blow
D
0.45%
mouse IgM
2.56%
HT2-280high
HT2-280
E
0.63%
0.71%
F
Lysotracker
Nuclei
Control-AOs (P0)
HPS2-AOs (P0)
cHPS2-AOs (P0)
G
HPS2-AOs (P2)
H
Lysotracker
FM1-43
Merged
Phase Contrast
30 sec
330 sec
HPS2-AO(P0)-Epithelium (Normal)
I
n.s
FM1-43 augumentation (Arbitrary Unit)
2.0
1.5
1.0
1.5
0
Normal
Enlarged
HPS2-AO(P0)-Epithelium

Table S1. Predicted off-target sites for CRISPR-sgRNA, Related to Figure 1.

chromosome	strand	start	end	sequence
chr3	+	85269952	85269972	CGTTT--ACAGGCAAAATAATGG
chr4	+	84242401	84242422	AGTTTAAAC-GTCAAAATAATGG
chr5	+	122400128	122400150	GGTATAGACAGGCAAAACAATGG
chr6	+	30393163	30393185	GGTTTATAA-AAGCAAAATAATGG
chr6	+	60699704	60699726	GGATTAAATAGTCAAAATAATGG
chr6	+	101860608	101860628	GGTCT-AACAGGCAAAATAA-GG
chr6	+	144457237	144457260	GGTTTAAAGAGGCAAAATGAATGG
chr6	+	152250503	152250524	GGTCTAAA-AAGCAAAATAATGG
chr6_GL000250v2_alt	+	1722659	1722681	GGTTTATAA-AAGCAAAATAATGG
chr6_GL000251v2_alt	+	1872847	1872869	GGTTTATAA-AAGCAAAATAATGG
chr6_GL000252v2_alt	+	1649013	1649035	GGTTTATAA-AAGCAAAATAATGG
chr6_GL000253v2_alt	+	1703400	1703422	GGTTTATAA-AAGCAAAATAATGG
chr6_GL000254v2_alt	+	1737213	1737235	GGTTTATAA-AAGCAAAATAATGG
chr6_GL000255v2_alt	+	1648248	1648270	GGTTTATAA-AAGCAAAATAATGG
chr6_GL000256v2_alt	+	1691623	1691645	GGTTTATAA-AAGCAAAATAATGG
chr7	+	3678866	3678887	GGTATCAACAGGCAAAATAAT-G
chr7	+	8542826	8542847	AGTTTACACAGGCAAAATAAT-G
chr9	+	84293388	84293408	GGTTTAAACAGG--AAATTATGG
chr9	+	98542216	98542237	GGTTTAAAAATGCAAAATAA-GG
chr9	+	99984208	99984229	GGTTT-TATAGGCAAAATAATGG
chr9	+	112715180	112715200	GGTTTAAA-AGACAAAA-AATGG
chr10	+	73652954	73652976	GGTTCAAACA-GCAAAAATAATGG
chr10	+	102727399	102727422	GGTTTAAACAGGCAGGAAAT-ATGG
chr11	+	119514483	119514505	TGTTAAAAGAGGCAAAATAATGG
chr12	+	130173993	130174015	TGTTAAAACAGACAAAATAATGG
chr16	+	83854456	83854477	GGTTT-AATAGGCAAAAGAATGG
chr21	+	32070464	32070483	GGTTTAAACA-GCAAAAT-AT-G
chr21	+	34916634	34916654	GGTTTAGACAGGC-AAA-AATGG
chrX	+	24881839	24881860	GGTGAAAACAGGCAAAAT-ATGG
chrX	+	96805068	96805091	GGTTTAATATAGGCAAAATAATGG
chrY	+	11502526	11502547	GGATTAAACAGG-AAAAAAATGG
chr1	-	61652206	61652227	GC-TTATTTTTCCTGTTTAAACC
chr1	-	152893492	152893511	CCATTATTTTG-CT-TTT-AACC
chr2	-	15325113	15325137	CCATTATTTTGCCTTTTTGTAAACC
chr2	-	124743782	124743803	CCATTTTTTTTCCTG-TTAAACC
chr2	-	150131432	150131453	CCATTTATTTT-CCTGTTTAAA-C
chr2	-	199174432	199174452	CCATT-TTTTACCT-TTTAAACC
chr3	-	27411420	27411440	CCATTATTTT-CCT-TTTAAAGC

chr3	-	70108850	70108870	CAATT-TTTTG-CTGTTTAAACC
chr4	-	3013664	3013684	CCTTTATTTTG-CTGTTTAAA-C
chr4	-	20408383	20408405	CCATTAATTTGTCTGTTTGAACC
chr5	-	92717234	92717254	CCA-T-TTTTGCCTGTTTATACC
chr6	-	83994041	83994063	CCTTTCTTTTTCCTGTTTAAACC
chr6	-	147566614	147566637	CAATTATTTTGCCAGGTTTAAACC
chr6	-	163497336	163497357	CAATTATTTTG-CTGTTTAAAAC
chr7	-	102818353	102818375	ACATTATTTAACCTGTTTAAACC
chr7	-	121948701	121948722	CCATTATTTTG-TTATTTAAACC
chr8	-	135459209	135459230	CCAGT-TTTTGCCTGTTTCAACC
chr10	-	11548921	11548940	CCATTA-TTT-CCT-TTTAAACC
chr10	-	21218346	21218368	TCTTTAATTTGCCTGTTTAAACC
chr10	-	23130755	23130775	CCATTATTTCGCCTG-TTAAA-C
chr12	-	89207482	89207504	CCATTATTTTTCCTATTTAAAAC
chr13	-	45752101	45752123	CCATTATTTTGCCTGTTTTGTCC
chr13	-	95785431	95785452	TCATTATTATGCCT-TTTAAACC
chr17	-	5432517	5432538	TAATTATTTT-CCTGTTTAAACC
chr17	-	60139533	60139553	CCGTTATTTTGCCT-TTT-AACC
chrX	-	14360143	14360164	CCTTTCTTTTGCCTG-TTAAACC
chrX	-	88375144	88375165	TCTTTATTTTGCCT-TTTAAACC

Table S2. Antibodies used in the present study. Related to Figures 1-4 and S1-3.

Species	Antibodies	Company	Catalog No./ Clone	Dilution
For isolating NKX2-1^{+} cells				
mouse	Anti-CPM	Abcam	ab49278/1C2	1:200
mouse	Anti-CPM	Wako	014-27501	1:200
donkey	Anti-mouse IgG- Alexa Fluor 647	Thermo Fisher Scientific	A-31571	1:200
mouse	Anti-CD47-FITC	BioLegend	323106/CC2C6	1:20
mouse	Anti-CD26-APC	BioLegend	302710/BA5b	1:20
mouse	Mouse IgG1-kappa-FITC	BD Pharmingen	555748/MOPC-21	1:5
mouse	Mouse IgG2a-kappa-APC	BD Pharmingen	550882/G155-178	1:20
For CD63 analysis				
mouse	Anti-CD63- Alexa Fluor 647	BioLegend	353016/H5C6	1:20
mouse	Mouse IgG1 kappa- Alexa Fluor 647	BioLegend	400130/MOPC-21	1:20
For passage of AOs by MX35				
mouse	Anti-NaPi2b	kindly provided by Dr. Gerd Ritter (MX35)		1:100
mouse	Mouse IgG1	Sigma-Aldrich	M5284/MOPC21	1:100
goat	Anti-EpCAM	R&D systems	AF960	1:100
donkey	Anti-mouse IgG Alexa Fluor 647	Thermo Fisher Scientific	A-31571	1:200
donkey	Anti-goat IgG Alexa Fluor 488	Thermo Fisher Scientific	A-11055	1:200
For isolation of adult human AT2 cells				
mouse	Anti-EpCAM	BioLegend	324202/9C4	1:100
mouse	Mouse IgG2b kappa	Thermo Fisher Scientific	14-4732-82/ eBMG2b	1:100
mouse	Anti-CD45-VioBlue	Miltenyi Biotec	130-092-880/5B1	1:100
mouse	Mouse IgG2a-VioBlue	Miltenyi Biotec	130-098-898/ S43.10	1:100
mouse	Anti-NaPi2b	kindly provided by Dr. Gerd Ritter (MX35)		1:100
mouse	Mouse IgG1	Sigma-Aldrich	M5284/MOPC21	1:100
mouse	HT2-280	Terrace Biotech	TB-27AHT2-280	1:100
mouse	Mouse IgM	Sigma-Aldrich	M5909/MOPC104E	1:100
goat	Anti-mouse IgG2b Alexa Fluor 488	Thermo Fisher Scientific	A-21141	1:200
goat	Anti-mouse IgG1 Alexa Fluor 647	Thermo Fisher Scientific	A-21240	1:200
donkey	Anti-mouse IgM Alexa Fluor 647	Jackson ImmunoResearch	715-605-140	1:50
FM1-43 augmentation assay				
mouse	Anti-EpCAM	Santa Cruz Biotechnology	sc-66020/EBA-1	1:100
rat	Anti-mouse IgG1-microbeads	Miltenyi Biotec	130-047-101	1:5
For immunofluorescence staining				
goat	Anti-β3A subunit (AP3B1)	abcam	AB118584	1:100
rabbit	Anti-SFTPC	Santa Cruz Biotechnology	FL-197	1:100
mouse	Anti-NaPi2b	kindly provided by Dr. Gerd Ritter (MX35)		1:100
rabbit	Anti-ABCA3	kindly provided by Prof. Nobuya Inagaki		1:500
mouse	Anti-DCLAMP	Beckman Coulter	IM3448/104.G4	1:100

rabbit	Anti-NKX2-1	abcam	ab76013/EP1584Y	1:500
mouse	Anti-SSEA-4	BioLegend	330401/MC-813-70	1:100
rabbit	Anti-SOX2	Millipore	AB5603	1:500
mouse	Anti-OCT3/4	Santa Cruz Biotechnology	sc-5279/C-10	1:100
rabbit	Anti-NANOG	ReproCELL	RCAB004P-F	1:100
donkey	Anti-rabbit IgG-Cy3	Jackson ImmunoResearch	711-165-152	1:500
donkey	Anti-mouse IgG-Alexa Fluor 488	Thermo Fisher Scientific	A-21202	1:500
donkey	Anti-goat IgG-Alexa Fluor 488	Thermo Fisher Scientific	A-11055	1:500
For Western blotting				
rabbit	Anti-β3A subunit (AP3B1)	Proteintech	13384-1-AP	1:500
rabbit	Anti-μ subunit (AP3M1)	Novus Biologicals	NBP1-76589	1:1000
rabbit	Anti-μ subunit (AP3M1)	Novus Biologicals	NBP2-27068	1:1000
mouse	Anti-β-actin	Sigma-Aldrich	A2228/AC-74	1:5000
pig	Anti-rabbit immunoglobulins-HRP	Dako	P0399	1:1000

Table S3. Content of the mediums. Related to Figures 2 and 4.

	Anteriorization medium	Ventralization medium	CFKD preconditioning medium
Basal Medium	DMEM / F12<br>Glutamax<br>B27 supplement (2 %)<br>L-ascorbic acid (0.05 mg/ml)<br>monothioglycerol (0.4 mM)<br>Penicillin / Streptomycin (50 U/ml)		
Chemicals / Cytokines	Noggin (100 ng/ml)<br>SB431542 (10 μM)	ATRA (depends on cell lines)<br>BMP4 (20 ng/ml)<br>CHIR99021 (depends on cell lines)<br>[Concentrations of ATRA/CHIR99021]<br>201B7: 0.05 μM/3.0 μM<br>409B2: 0.1 μM/2.5 μM<br>604A1: 1.0 μM/2.5 μM<br>H9: 0.5 μM/3.5 μM<br>HPS2- and cHPS2-iPSCs: 1.0 μM/3.5 μM	CHIR99021 (3 μM)<br>FGF10 (10 ng/ml)<br>KGF (10 ng/ml)<br>DAPT (20 μM)

	Alveolarization medium	Replating medium
Basal Medium	Ham's F12<br>B27 supplement (1 %)<br>BSA (0.25 %)<br>HEPES (15 mM)<br>$CaCl_2$ (0.8 mM)<br>ITS premix (0.1 %)<br>Penicillin / Streptomycin (50 U/ml)	
Chemicals / Cytokines	Dexamethasone (50 nM)<br>IBMX (100 μM)<br>KGF (10 ng/ml)<br>8-Br-cAMP (100 μM)	Dexamethasone (50 nM)<br>IBMX (100 μM)<br>KGF (10 ng/ml)<br>CHIR99021 (3 μM)<br>SB431542 (10 μM)

Table S4. Primers used in the present study. Related to Figures 1-2 and S2.

Gene	Forward	Reverse	Size
ABCA3	TCTCCTTCAGCTTCATGGTCAG	TGGCTCAGAGTCATCCAGTTG	136
ACTA2	GGGTGACGAAGCACAGAGCA	CTTCAGGGGCAACACGAAGC	137
AGER	GCCACTGGTGCTGAAGTGTA	TGGTCTCCTTTCCATTCCTG	191
AP3B1	GAAGAATGCAGCCCATGCAA	TGCCAGCTACCAATGTGCTT	116
AQP5	CTGTCCATTGGCCTGTCTGTC	GGCTCATACGTGCCTTTGATG	248
FOXJ1	CCTGTCGGCCATCTACAAGT	AGACAGGTTGTGGCGGATT	94
KRT5	GAGCTGAGAAACATGCAGGA	TCTCAGCAGTGGTACGCTTG	82
MUC5AC	CATCTGCCAGCTGATTCTGA	AAGACGCAGCCCTCATAGAA	129
PDPN	TCCAGGAACCAGCGAAGAC	CGTGGACTGTGCTTTCTGA	119
SCGB1A1	CACCATGAAACTCGCTGTCAC	AGTTCCATGGCAGCCTCATAAC	147
SFTPB	GAGCCGATGACCTATGCCAAG	AGCAGCTTCAAGGGGAGGA	133
SFTPC	GCAAAGAGGTCCTGATGGAG	TGTTTCTGGCTCATGTGGAG	178
SLC34A2	TCGCCACTGTCATCAAGAAG	CTCTGTACGATGAAGGTCATGC	112
SNAIL	AATCCAGAGTTTACCTTCCAGCA	TCCCAGATGAGCATTGGCAG	110
TGFB1	GGAAATTGAGGGCTTTCGCC	CCGGTAGTGAACCCGTTGAT	90
TP63	ACTGCCAAATTGCAAAGACA	TGACTAGGAGGGGCAATCTG	184
TWIST	TCTCGGTCTGGAGGATGGAG	AATGACATCTAGGTCTCCGGC	100
β-ACTIN	CAATGTGGCCGAGGACTTTG	CATTCTCCTTAGAGAGAAGTGG	126

SUPPLEMENTAL EXPERIMENTAL PROCEDURES

Generation of human iPSCs generation and maintenance of human PSCs

For the generation of HPS2-iPSCs, GM17890 HPS2 fibroblasts (NIGMS Human Genetic Cell Repository, Coriell Institute for Medical Research) were reprogrammed to iPSCs as previously reported (Okita et al., 2011). Human cDNAs of reprogramming factors were transduced into patient fibroblasts with episomal vectors (SOX2, KLF4, OCT3/4, L-MYC, LIN28, short hairpin RNA for p53). Several days after transduction, fibroblasts were harvested and replated on mitomycin C-treated STO feeder layer (DS Pharma Biomedical). The next day, the medium was changed to primate ES cell medium (Reprocell) supplemented with human FGF-2 (4 ng/ml) (Wako or DS Pharma Biomedical). The medium was then changed every other day. Thirty days after transduction, the iPSC colonies were picked up.

For the teratoma formation assay, undifferentiated HPS-iPSCs were harvested and re-suspended in DMEM/F12 supplemented with 10 μM of Y-27632 (LC laboratories). Approximately 5 x 10^5 cells were injected into a testis of 8-week-old SCID mice (Clea Japan, Inc.). At 10-12 weeks after transplantation, the mice were sacrificed, and tumors were harvested and fixed with Mildform 10N (Wako) and embedded in paraffin. The sections were stained with hematoxylin and eosin.

For the maintenance of H9 human embryonic stem cells (hESCs), the cells were cultured on mitomycin C-treated STO feeder cells in Primate ES medium supplemented with 4 ng/ml human FGF-2 and 50 U/ml penicillin–streptomycin (Life Technologies). HPS2-iPSCs (GM17-6), cHPS2-iPSCs (res69-5) and control iPSCs (201B7, 409B2 and 604A1) were cultured in Essential 8 medium (Gibco) without feeder cells and used for differentiation studies within 20 passages.

Gene correction of HPS2-iPSCs

The donor template was designed to have normal exon 18. Since AP3B1 is expressed ubiquitously, the splicing acceptor was placed upstream of the neomycin resistant gene in the donor template. HPS2-iPSCs were pretreated with Y-27632 at 10 μM for at least 1 h before electroporation. The cells were washed with PBS and treated with CTK solution for 1–3 min at room temperature (RT) to remove feeders and then washed with PBS twice. Next, the iPSCs were treated with accutase for 10 min at 37°C, dissociated into single cells by pipetting, and neutralized with culture medium. We electroporated 10 μg of Cas9 and sgRNA expression vectors (5 μg for Cas9 and 5 μg for sgRNA) and 5 μg of donor plasmid into 1 × 10^6 cells using a NEPA 21 electroporator (poring pulse: pulse voltage, 125 V; pulse width, 5 ms; pulse number, 2; Nepagene). Cells were plated onto one well of a six-well plate with feeders in the presence of 10 μM Y-27632 for 1–2 days. G418 (Gibco) selection was applied after iPSC colonies were recovered (4–5 days after transfection). The resulting neomycin-resistant colonies were dissociated into single cells and plated at 200, 500 and 1500 cells per 10-cm dish with feeders. Each subclone was screened by genomic PCR and sequencing. After establishing the knockin clones, we electroporated the cells with 10 μg of the Cre expression vector using NEPA 21. Clone isolation was carried out and excision of the neomycin-selection cassette was confirmed by genomic PCR and sequencing.

Out of 132 isolated clones, 94 morphologically normal clones were screened for knock-in; 36 clones (27%) had the donor template at the target locus. After Cre excision, we chose a res69-5 clone for the subsequent experiments. For res69-5 clone, a total of 58 predicted off-target sites with up to 3 bp mismatches allowed (Table S1) (GGGenome; https://gggenome.dbcls.jp/ja/) were examined by sanger sequencing, resulting in no abnormal deletions or insertions being detected at these sites in the res69-5 cHPS2-iPSC clone.

Western blotting

Thirty micrograms of protein in lysis buffer was loaded in each well of 10% polyacrylamide gels and electrophoresed at 100 V for 2 h. Proteins were transferred onto methanol-activated PVDF membrane (Amersham Hybond P PVDF 0.45) in transfer buffer

(20% methanol, 0% SDS) at 100 V for 1 h at 4°C. After being washed in TBS-T for 5 min, membranes were blocked with TBS-T containing 5% non-fat milk (Megmilk Snow Brand) for 1 h under agitation. Primary and secondary antibodies were diluted in TBS-T. Primary antibodies were incubated overnight and Secondary antibodies were incubated for 1 h under agitation, followed by washing with TBS-T for 5 min, three times. Signals were visualized by Clarity Western ECL Substrate (Bio-rad). The antibodies used are listed in Table S2.

FACS analysis

Single cell suspension was washed with FACS buffer (1% BSA /PBS supplemented with 10 μM Y-27632) and immunostained with primary antibodies at 4°C for 15 min. After being washed twice with FACS buffer, the cells were stained with secondary antibodies on demand at 4°C for 15 min. After being washed twice with FACS buffer, the cells were stained with propidium iodide (PI), and a FACS analysis was performed using a FACS Aria II or Aria III (BD Biosciences). The antibodies used are listed in Table S2.

Induction and isolation of NKX2-1^{+} lung progenitor cells.

The differentiation of human PSCs was performed as previously described (Gotoh et al., 2014; Konishi et al., 2016; Yamamoto et al., 2017). In brief, undifferentiated human PSCs were differentiated into definitive endodermal cells on Geltrex (Gibco)-coated plates in RPMI medium (Nacalai Tesque) containing a saturated dose of Activin A (100 ng/ml) (Peprotech), 1 μM CHIR99021 (Axon Medchem), 2% B27 supplement (Life technologies) and 50 U/ml penicillin–streptomycin. The medium was replaced every two days. Y-27632 was supplemented on Day 0, and sodium butyrate were supplemented on Day 1, 2 and 4. During Day 6-10, the definitive endodermal cells were cultured in the anteriorization medium (Table S3), followed by switching on Day10 to the ventralization medium (Table S3) containing BMP4 (20 ng/ml) (HumanZyme) and adjusted doses of ATRA (Sigma-Aldrich)/CHIR99021 (Table S3). The optimized concentrations of ATRA/CHIR99021 for both HPS2-iPSCs (GM17-6) and cHPS2-iPSCs (res69-5) were 1.0 μM/3.5 μM. During Day 14-21, cells were cultured in CFKD preconditioning medium (Table S3). On Day 21, NKX2-1^{+} lung progenitor cells were isolated using mouse anti-human CPM (Abcam or Wako) and anti-mouse IgG-Alexa647 (Invitrogen) for gating CPM^{high} cells as previously reported (Yamamoto et al., 2017). To validate CD47/CD26-based isolation, mouse anti-human CD47-FITC (BioLegend) and mouse anti-human CD26-APC (BioLegend) were used for gating CD47^{high} or CD47^{high}CD26^{low} cells. Mouse IgG1-FITC (BD Pharmingen) and mouse IgG2a-APC (BD Pharmingen) were used for isotype controls. The antibodies used are listed in Table S2.

AO formation

A total of 1.0 × 10^{4} CPM^{high} cells and 5.0 × 10^{5} human fetal lung fibroblasts were mixed in 100 μL of alveolarization medium (Table S3) supplemented with Y-27632 (10 μM) and 100 μL of Matrigel (Corning; 354230) and placed on a 12-well cell culture insert (Corning). The medium in the lower chamber was changed every two days. Human fetal lung fibroblasts (17.5 weeks of gestation; DV Biologics; PP002-F-1349, lot 121109VA) were cultured in DMEM (Nacalai Tesque) supplemented with 10% fetal bovine serum and used at passage number 9 or 10.

AO passaging

The duration of organoid culturing was 14±1 days. AO cells were dissociated with 0.1% Tripsin-EDTA at 37 °C for 15 minutes (Gotoh et al., 2014; Yamamoto et al., 2017), washed in FACS buffer twice, and immunostained with mouse anti-NaPi2b (MX35; kindly provided by Dr. Gerd Ritter) (Yin et al, 2008) and goat anti-EpCAM (R&D Systems). Anti-mouse IgG-Alexa647 (Invitrogen) and anti-goat IgG-Alexa488 (Invitrogen) were used as secondary antibodies. NaPi2b^{high} and NaPi2b^{low} cell populations were then isolated by FACS Aria II or III (BD Biosciences). A total of 1.0 × 10^{4} NaPi2b^{high} cells were mixed with 5.0

× 10^5 human fetal lung fibroblasts in Matrigel and placed on a culture insert as described in AO formation. The antibodies used are listed in Table S2.

qRT-PCR

Total RNA was extracted with a PureLink RNA mini kit (Invitrogen). cDNA was prepared from 80 ng of total RNA per sample with SuperScript III reverse transciptase (Invitrogen), amplified by Power SYBR Green PCR Master Mix (Applied Biosystems) and quantified by QuantStudio 3 (Applied Biosystems). The gene expression was normalized to the β-actin expression and compared with that in control RNA of human fetal lung at 17, 18 and 22 weeks of gestation (Agilent Technologies; #540177, lot 0006055802) and was presented as the relative gene expression. The primers used in the present study are described in Table S4.

IF staining

Two-dimensional culture or slide samples were fixed with 4% paraformaldehyde/PBS for 15 min and permeabilized with 0.2% Triton X-100/PBS for 15 min. After 30 min of blocking in 5% normal donkey serum/1% BSA/PBS, they were immunostained with primary and secondary antibodies for 30 min each, as previously described (Gotoh et al, 2014). In contrast, AOs were fixed with 4% paraformaldehyde/PBS for 20 min and incubated in 30% sucrose/PBS overnight. Organoids were then embedded in OCT compound (Sakura Finetek) and frozen in liquid nitrogen. The frozen organoids were sliced into 10-μm-thick sections on slides, permeabilized and blocked as described above. They were then immunostained with primary antibodies overnight and with secondary antibodies for 1 h. Hoechst-33342 (Dojindo) was added to the secondary antibody solution in order to label nuclei. The antibodies used in the present study are listed in Table S2. For the quantitative analysis of IF imaging, images were taken by a BZ-X710 (Keyence) and quantified by Hybrid Cell Count/BZ-H3C (Keyence) from 10-15 areas. A TCS SP8 (Leica Microsystems) was used for confocal imaging, and an IX81 (Olympus) was used for other conventional IF imaging.

Isolation of adult human AT2 cells from lung tissues

Human AT2 cells were isolated as previously described, with some modifications (Fujino et al., 2012; Yamamoto et al., 2017). Lung tissues were obtained from nontumorous regions of surgically resected specimens from patients with lung tumors. Samples from 3 donors (77-year-old male, 64-year-old female, 62-year-old female) were used for the cytospin analysis and 4 donors (58-year-old female, 76-year-old male, 72-year-old female, 68-year-old male) were used for the qRT-PCR. Lung tissues were mechanically minced and dissociated with Dispase II (Wako), collagenase/Dispase (Sigma-Aldrich) and DNase I (Sigma-Aldrich) for 60 min at 37°C. After being filtered through a 100-μm mesh, the cells were treated with ACK lysing buffer (Gibco). For sorting with MX35 (anti-NaPi2b), cells were immunostained with primary antibodies of MX35, anti-human EpCAM and anti-human CD45-VioBlue and secondary antibodies of anti-mouse IgG2b-Alexa488 (Invitrogen) and anti-mouse IgG1-Alexa647 (Invitrogen). For sorting with HT2-280, HT2-280 (Terrace Biotec) and anti-mouse IgM-Alexa647 (Jackson ImmunoResearch) were used instead of MX35 and anti-mouse IgG1-Alexa 647, respectively. The antibodies and corresponding isotype controls used in the present study are listed in Table S2.

Electron microscopy

Small pieces of AOs were incubated in fixative solution consisting of 2.5% glutaraldehyde, 2% paraformaldehyde, 2% osmium tetroxide, 0.1% picric acid, 4% sucrose, and 0.1M phosphate buffer (pH 7.4) on ice for 2 h. *En bloc* staining was performed in 1% uranyl acetate at RT for 1 h, dehydrated, and embedded in Epon 812, as described previously (Gotoh et al., 2014). Ultrathin sections were stained with uranyl acetate and lead citrate and analyzed by transmission electron microscopy (Hitachi; H-7650).

Live cell imaging

AOs were stained with 10 µg/ml Hoechst 33342 and 100 nM Lysotracker Red DND-99 (Thermo Fisher Scientific; L7528) for 60 and 30 min, respectively. AOs were washed with PBS twice, placed on 35 mm glass-bottom dishes (Grainer) and examined under a FV10i-LIV confocal microscope (Olympus) under 5% CO_2 at 37°C. The apicobasal distribution of the Lysotracker-stained vesicles was quantified using the Image J software program (National Institutes of Health).

For the LB secretion assay, AOs were dissociated with 0.1% Tripsin-EDTA at 37°C for 15 min and stained with anti-EpCAM (Santa Cruz Biotechnology) followed by staining with anti-mouse IgG1-microbeads (Miltenyi Biotec), followed by separation using a LS column (Miltenyi Biotec). To obtain a sufficient number of cells, $EpCAM^+$ cells were used for the experiment. A total of 3.0×10^5 $EpCAM^+$ cells were reseeded on Geltrex-coated 8-well coverglass chambers (Thermo Fisher Scientific) in replating medium (Table S3) supplemented with 10 µM of Y-27632. At 24 ± 12 h after reseeding, cells were stained with Lysotracker Red DND-99 (100 nM) for 30 min, washed with PBS and given fresh replating medium. Before live cell imaging, the cells were incubated in 0.5 µM of FM1-43 (Thermo Fisher; T3163). Time-lapse imaging was then performed after the supplementation of the secretagogue cocktail prepared as stock solutions of forskolin (Dekkers et al., 2013), ATP (Haller et al., 1998), ionomycin (Neuland et al., 2014) and phorbol 12-myristate 13-acetate (PMA) (Neuland et al., 2014). The LB content secretion was quantified by calculating the FM1-43 augmentation level as “the change in the FM1-43 integral density divided by the change in the Lysotracker integral density”. For the comparison of enlarged and normal LBs in HPS2-AO, LBs of>4 µm in diameter were analyzed as “enlarged”; LBs of ≤4 µm in diameter were analyzed as “normal”. Image J was used to analyze images. Z-stack images were obtained before and 60 min after stimulation with the secretagogue cocktail. Lysotracker Deep Red (Thermo Fisher Scientific; L12492) (100 nM) was used for Z-stack images.

PC assay

A total of 3.0×10^5 $EpCAM^+$ cells were reseeded on a 96-well flat bottom plate in replating medium (Table S3) supplemented with 10 µM of Y-27632. At 24 ± 12 h after reseeding, the medium was removed and replaced with 200 µl of HBSS containing secretagogue cocktail or DMSO. After 3 hours, culture supernatant was collected. A phosphatidylcholine assay kit (Sigma-Aldrich; MAK049) was used for the quantification.

SUPPLEMENTAL REFERENCES

Dekkers, J.F., Wiegerinck, C.L., de Jonge, H.R., Bronsveld, I., Janssens, H.M., de Winter-de Groot, K.M., Brandsma, A.M., de Jong, N.W., Bijvelds, M.J., Scholte, B.J., et al. (2013). A functional CFTR assay using primary cystic fibrosis intestinal organoids. Nat Med 19, 939-945.

Fujino, N., Kubo, H., Ota, C., Suzuki, T., Suzuki, S., Yamada, M., Takahashi, T., He, M., Suzuki, T., Kondo, T., et al. (2012). A novel method for isolating individual cellular components from the adult human distal lung. Am J Respir Cell Mol Biol 46, 422-430.

Neuland, K., Sharma, N., and Frick, M. (2014). Synaptotagmin-7 links fusion-activated Ca^{2+} entry and fusion pore dilation. J Cell Sci 127, 5218-5227.

Okita, K., Matsumura, Y., Sato, Y., Okada, A., Morizane, A., Okamoto, S., Hong, H., Nakagawa, M., Tanabe, K., Tezuka, K., et al. (2011). A more efficient method to generate integration-free human iPS cells. Nat Methods 8, 409-412.
