# Supplementary material for: In Vitro Disease Modeling of Hermansky-Pudlak Syndrome Type 2 Using Human Induced Pluripotent Stem Cell-Derived Alveolar Organoids
Source: Stem Cell Reports. 2019 Feb 14;12(3):431–40. doi: 10.1016/j.stemcr.2019.01.014 (PMC6409438; doi:10.1016/j.stemcr.2019.01.014)
Supplement: Document S2. Article plus Supplemental Information [file mmc3.pdf]

Stem Cell Reports
Report

OPEN ACCESS

# *In Vitro* Disease Modeling of Hermansky-Pudlak Syndrome Type 2 Using Human Induced Pluripotent Stem Cell-Derived Alveolar Organoids

Yohei Korogi,[1] Shimpei Gotoh,[1,2,*] Satoshi Ikeo,[1] Yuki Yamamoto,[1] Naoyuki Sone,[1] Koji Tamai,[1] Satoshi Konishi,[1] Tadao Nagasaki,[1] Hisako Matsumoto,[1] Isao Ito,[1] Toyofumi F. Chen-Yoshikawa,[3] Hiroshi Date,[3] Masatoshi Hagiwara,[4] Isao Asaka,[5] Akitsu Hotta,[6] Michiaki Mishima,[1] and Toyohiro Hirai[1]

[1]Department of Respiratory Medicine, Graduate School of Medicine, Kyoto University, Kyoto 606-8507, Japan
[2]Department of Drug Discovery for Lung Diseases, Graduate School of Medicine, Kyoto University, Kyoto 606-8501, Japan
[3]Department of Thoracic Surgery, Graduate School of Medicine, Kyoto University, Kyoto 606-8507, Japan
[4]Department of Anatomy and Developmental Biology, Graduate School of Medicine, Kyoto University, Kyoto 606-8501, Japan
[5]Department of Fundamental Cell Technology, Center for iPS Cell Research and Application, Kyoto University, Kyoto 606-8507, Japan
[6]Department of Clinical Application, Center for iPS Cell Research and Application, Kyoto University, Kyoto 606-8507, Japan
*Correspondence: a0009650@kuhp.kyoto-u.ac.jp
https://doi.org/10.1016/j.stemcr.2019.01.014

## SUMMARY

It has been challenging to generate *in vitro* models of alveolar lung diseases, as the stable culture of alveolar type 2 (AT2) cells has been difficult. Methods of generating and expanding AT2 cells derived from induced pluripotent stem cells (iPSCs) have been established and are expected to be applicable to disease modeling. Hermansky-Pudlak syndrome (HPS) is an autosomal recessive disorder characterized by dysfunction of lysosome-related organelles, such as lamellar bodies (LBs), in AT2 cells. From an HPS type 2 (HPS2) patient, we established disease-specific iPSCs (HPS2-iPSCs) and their gene-corrected counterparts. By live cell imaging, the LB dynamics were visualized and altered distribution, enlargement, and impaired secretion of LBs were demonstrated in HPS2-iPSC-derived AT2 cells. These findings provide insight into the AT2 dysfunction in HPS patients and support the potential use of human iPSC-derived AT2 cells for future research on alveolar lung diseases.

## INTRODUCTION

Alveolar type 2 (AT2) cells are tissue stem cells that maintain homeostasis of the alveolar region of the lung (Barkauskas et al., 2013). They secrete pulmonary surfactant to prevent alveolar collapse and contribute to the host defense of the lung (Whitsett et al., 2015). Lamellar bodies (LBs), characteristic organelles of mature AT2 cells, are lysosome-related organelles (LROs) involved in the storage and secretion of pulmonary surfactant and are often affected in alveolar lung diseases, including hereditary pulmonary fibrosis (PF) (Nakatani et al., 2000; Whitsett et al., 2015). However, the mechanism underlying the LB degeneration and AT2 cell dysfunction in human alveolar lung diseases is not well understood due to poor accessibility and the difficulty of isolating and culturing primary AT2 cells. Induced pluripotent stem cells (iPSCs) are expected to overcome these limitations. We previously established methods for generating human pluripotent stem cell (hPSC)-derived alveolar and airway cells in organoids (Gotoh et al., 2014; Konishi et al., 2016) and successfully expanded hPSC-derived AT2 cells in alveolar organoids (AOs) (Yamamoto et al., 2017).

Hermansky-Pudlak syndrome (HPS) is a rare autosomal recessive hereditary disease caused by mutations in the genes involved in the formation and maturation of LROs and characterized by oculocutaneous albinism and bleeding diathesis (El-Chemaly and Young, 2016). Among the ten subtypes, patients with HPS1, HPS2, and HPS4 can suffer from PF. HPS is a disease of LROs, and the abnormal enlargement of LBs in AT2 cells was reported in both HPS patients (Nakatani et al., 2000) and mouse models (Lyerla et al., 2003). HPS2 is an extremely rare subtype of HPS that is caused by mutations of the *AP3B1* gene, which encodes the β3A subunit of the AP-3 complex, which is involved in intracellular membrane traffic. It was previously reported that approximately 40% of HPS2 patients had PF and that 78% of HPS2 patients with PF were children (Jessen et al., 2013).

In this study, we generated HPS2 patient-derived iPSCs (HPS2-iPSCs) and gene-corrected iPSCs (cHPS2-iPSCs) and differentiated them into AOs (HPS2-AOs and cHPS2-AOs, respectively). Based on the comparison of these AOs, we report the AT2 cell dysfunction of HPS2-AOs.

## RESULTS

### Generation of HPS2-iPSCs and cHPS2-iPSCs

HPS2-iPSCs were established from patient fibroblasts obtained from the Coriell Institute for Medical Research (GM17890) (Figure 1A). The HPS2 patient donor had compound heterozygous nonsense mutations in exon 15 and 18 of the *AP3B1* gene and he was histologically diagnosed with nonspecific interstitial pneumonitis at 20 months of age (Huizing et al., 2002) (Figure 1B). Next, cHPS2-iPSCs were generated from HPS2-iPSCs by


This is an open access article under the CC BY license (http://creativecommons.org/licenses/by/4.0/).

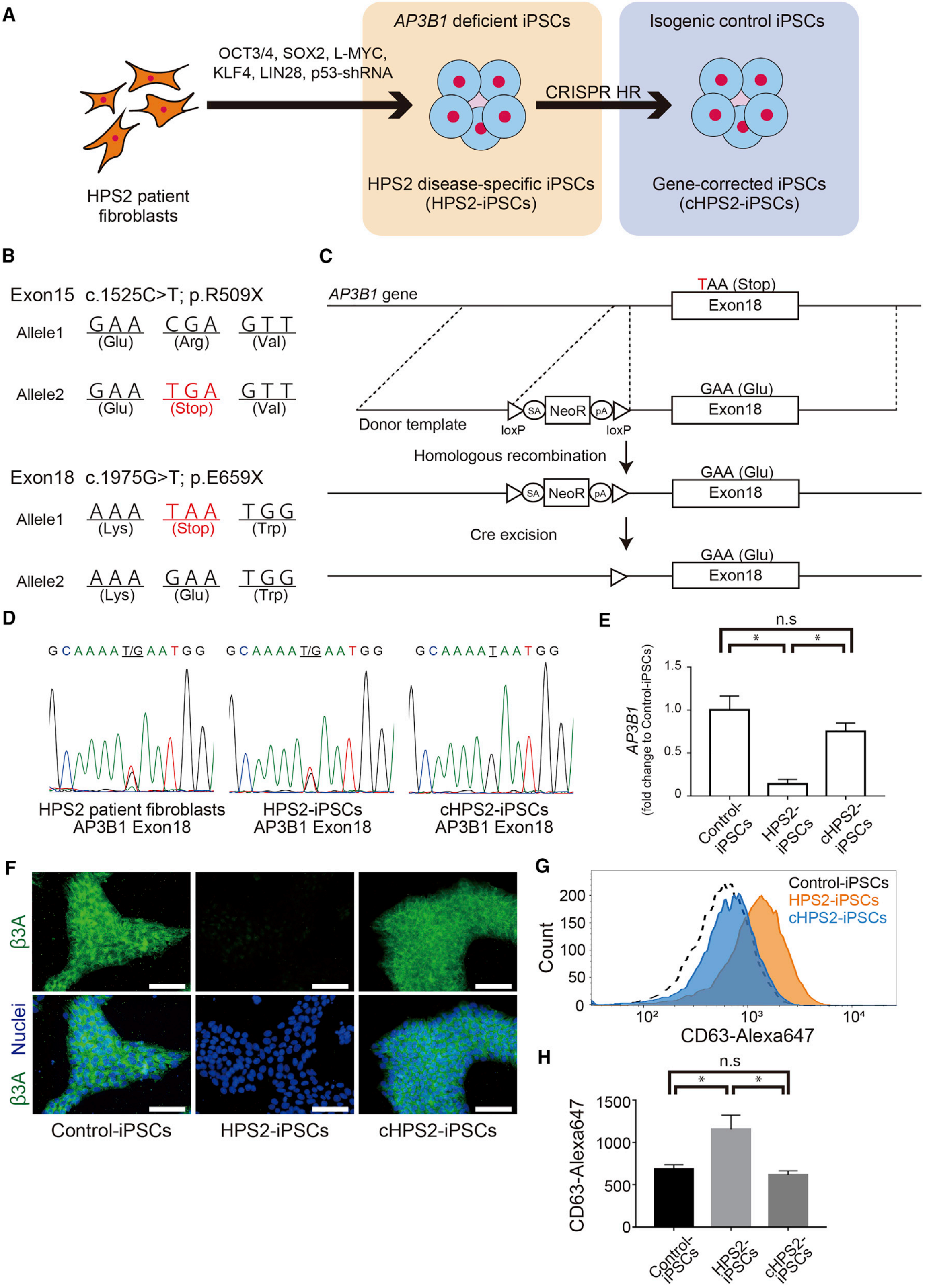


(legend on next page)

using CRISPR/Cas9-mediated homologous recombination (Li et al., 2015) (Figure 1C). We targeted the mutation on exon 18, because it was not possible to design a single guide RNA to hybridize with the mutation on exon 15. After G418 selection and limiting dilution, 36 out of 132 clones (27%) had the donor template at the target locus. After Cre excision, we chose a res69-5 clone for the subsequent experiments. The sequencing data showed that the mutation in exon 18 was corrected in cHPS2-iPSCs (Figures 1D and S1A). There were no indels at 58 predicted off-target sites (Table S1). The *AP3B1* transcript level was decreased to 14% ± 5% in HPS2-iPSCs and restored to 75% ± 10% in cHPS2-iPSCs, in comparison with normal control iPSCs (Figure 1E), which was indicative of nonsense-mediated mRNA decay (NMD) in HPS2-iPSCs, as reported in donor cells (Huizing et al., 2002). In immunofluorescence (IF) staining, the β3A subunit was almost absent in HPS2-iPSCs and was restored in cHPS2-iPSCs (Figure 1F). Western blotting demonstrated the absence of AP3B1 and the decrease of AP3M1 in HPS2-iPSCs, consistent with the previous report by Kook et al. (2018) (Figure S1B). Both HPS2-iPSCs and cHPS2-iPSCs expressed undifferentiated markers and showed no abnormal karyotypes (Figures S1C and S1D). The pluripotency was demonstrated by the teratoma formation (Figure S1E) and there was no integration of reprogramming vectors in genomic DNA (Figure S1F). CD63 molecules interact with AP-3 complex via its tyrosine-based targeting motif and are sorted to lysosomes (Rous et al., 2002). Since CD63 is mis-sorted to the cell surface in AP-3 dysfunction, the function of AP-3 complex is assayable by flow cytometry of CD63 (Dell'Angelica et al., 1999). In HPS2-iPSCs, the increased cell surface CD63 expression was observed in comparison with control iPSCs and cHPS2-iPSCs, suggesting the dysfunction of AP-3 complex in HPS2-iPSCs and its restoration in cHPS2-iPSCs (Figures 1G and 1H).

### Comparison of the Methods of NKX2-1$^+$ Cell Isolation

The isolation of NKX2-1$^+$ lung progenitor cells is a critical step in the generation of lung epithelial cells from hPSCs. We compared the isolation efficiency of previously reported sorting methods, carboxypeptidase M (CPM) and CD47 combined with or without CD26 (Figure S2A) (Gotoh et al., 2014; Hawkins et al., 2017). At day 21 of our induction protocol (Figure 2A), CPM$^{high}$ cells contained the most NKX2-1$^+$ cells in all hPSC lines (Figures S2B and S2C). Since there was no interaction between the cell lines and sorting methods analyzed by two-way ANOVA, the samples from different cell lines were all analyzed together. As a result, CPM-based sorting was able to isolate more NKX2-1$^+$ cells than CD47-based methods (Figure S2D).

### Induction of AOs from HPS2-iPSCs and cHPS2-iPSCs

Upon CPM-based sorting, NKX2-1$^+$ lung progenitor cells derived from both HPS2-iPSCs and cHPS2-iPSCs formed AOs after three-dimensional co-culture with human fetal lung fibroblasts (HFLFs). The expression levels of representative AT2 markers (*SFTPC*, *SFTPB*, and *ABCA3*) and alveolar type 1 (AT1) markers (*AQP5* and *AGER*) were not significantly different among the AOs derived from control hPSCs and HPS2-iPSCs and cHPS2-iPSCs (Figure 2B). The fibrosis-related markers (*TGFB1*, *ACTA2*, *SNAIL*, and *TWIST*) showed no differences between HPS2-AOs and cHPS2-AOs (Figure 2C), consistent with the findings of the animal study in that the *Ap3b1* mutant (Pearl) mice did not develop PF spontaneously (Young et al., 2007).

### Utility of NaPi2b as a Surface Antigen for the Subculture of AOs

NaPi2b encoded by *SLC34A2* is a multi-transmembrane protein expressed on AT2 cells, and MX35 is a monoclonal antibody that recognizes its extracellular domain (Yin et al., 2008). IF staining showed that SFTPC$^+$ cells in both adult and fetal human lung tissues expressed NaPi2b (Figure S2E). We investigated whether this antibody was useful for the isolation of AT2 cells from the adult human lung. The proportion of SFTPC$^+$ AT2 cells was 84.2% ± 6.5% in the NaPi2b$^{high}$ population and 84.8% ± 3.6% in the HT2-280$^{high}$ population (Figures S2F–S2I). qRT-PCR showed that the AT2 marker expression was concentrated in the

**Figure 1. Generation of HPS2-iPSCs and cHPS2-iPSCs**
(A) Schematic overview of the generation of HPS2-iPSCs and cHPS2-iPSCs.
(B) Different mutations in each allele of the patient fibroblasts.
(C) Strategy for correcting the mutation in exon 18.
(D) Sequence data of exon 18 in donor fibroblasts, HPS2-iPSCs, and cHPS2-iPSCs. The mutation was corrected in cHPS2-iPSCs.
(E) qRT-PCR of *AP3B1* in each cell line. 201B7 was used for control iPSCs (mean ± SEM, n = 3 independent experiments). A one-way ANOVA with Tukey's multiple comparisons test was used. *p < 0.05; n.s., not significant.
(F) IF staining of the β3A subunit of AP-3 complex in each iPSC line. 201B7 was used for control iPSCs. Scale bars, 100 μm.
(G) Surface CD63 expression in control iPSCs, HPS2-iPSCs, and cHPS2-iPSCs. 201B7 was used for control iPSCs.
(H) Median fluorescence intensity of CD63-Alexa647 (mean ± SEM, n = 3 independent experiments). A one-way ANOVA with Tukey's multiple comparisons test was used. *p < 0.05; n.s., not significant.
See also Figure S1.

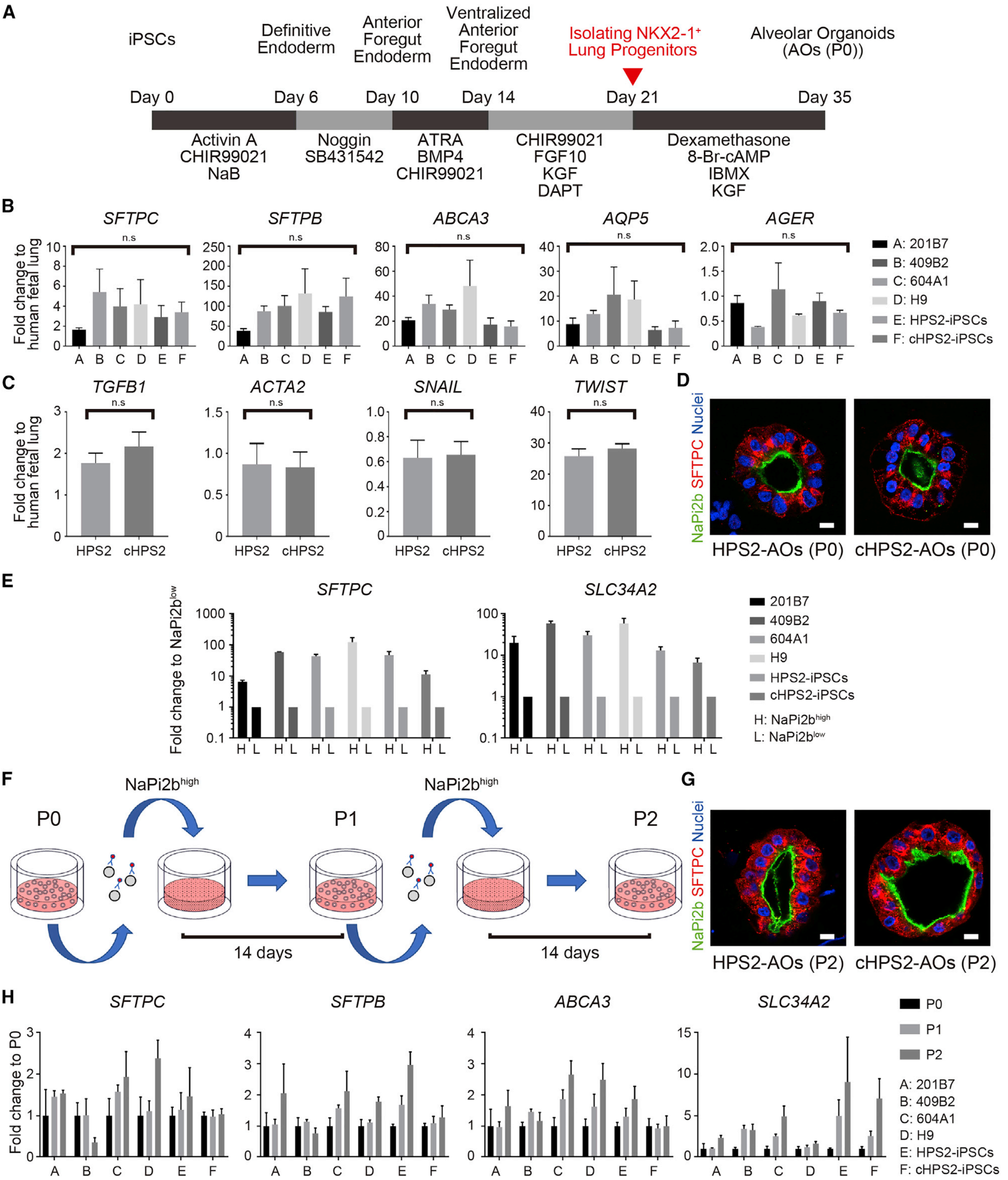


**Figure 2. Generation of iPSC-derived AOs and Their Expansion Based on the Expression of NaPi2b**

(A) Schematic overview of the generation of hPSC-derived AOs.

(B) qRT-PCR of AT1 and AT2 markers in AOs (P0) (mean ± SEM, n = 3 independent experiments). Kruskal-Wallis with Dunn's multiple comparisons test was used. n.s., not significant.

*(legend continued on next page)*

NaPi2b$^{high}$ population, whereas AT1 and airway markers were enriched in the NaPi2b$^{low}$ population, suggesting that AT2 cells were selectively isolated in the NaPi2b$^{high}$ population (Figures S2J).

NaPi2b was also expressed on the luminal surface of SFTPC$^{+}$ cells in hPSC-derived AOs regardless of the *AP3B1* genotype (Figure 2D). The NaPi2b$^{high}$ population contained more SFTPC$^{+}$ cells than the NaPi2b$^{low}$ population (Figures S3A–S3C), which was supported by the different levels of *SFTPC* and *SLC34A2* in both populations (Figure 2E). When NaPi2b$^{high}$ cells were co-cultured with HFLFs, AT2 cells were successfully subcultured in AOs, which also expressed AT2 cell markers with a stepwise increase of *SLC34A2* (Figures 2F–2H). In P2-AOs, NaPi2b was maintained on the luminal surface of SFTPC$^{+}$ cells in both HPS2-AOs and cHPS2-AOs, similarly to P0-AOs (Figure 2G). We also investigated HT2-280 for isolating AT2 cells in AOs, but the number of HT2-280$^{+}$ cells was too small for subculture or analysis (Figures S3D and S3E). These findings indicated that anti-NaPi2b monoclonal antibody (MX35) was more useful than HT2-280 for subculturing AT2 cells in hPSC-derived AOs.

### Abnormal Distribution and Aberrant Structures of LBs in HPS2-AOs

LBs in AOs were examined via live cell imaging using LysoTracker (LT), a fluorescent probe used for visualizing LBs (Haller et al., 1998; Yamamoto et al., 2017). Interestingly, whereas the LT$^{+}$ organelles were gathered on the apical side of the constituent epithelial cells in control AOs and cHPS2-AOs, they were distributed randomly in HPS2-AOs (Figures 3A–3C). This distributional difference was more clearly observed in P2-AOs than in P0-AOs (Figures 3A and S3F). In addition, the LT$^{+}$ organelles in HPS2-AOs seemed enlarged in P2-AOs (Figures 3A and 3B). In IF staining, ABCA3, a limiting membrane protein of the LBs (Yamano et al., 2001), was located on apically distributed vesicles in DCLAMP$^{+}$ cells of cHPS2-AOs whereas it was located on the enlarged and randomly distributed vesicles in those of HPS2-AOs, suggesting that LT$^{+}$ organelles corresponded to LBs (Figure 3D). In electron microscopy, giant LBs (>5 μm in diameter) were occasionally observed in HPS2-AOs, whereas the LBs in control AOs or cHPS2-AOs were of a normal size (1–2 μm in diameter) (Figure 3E). Furthermore, abnormally formed lysosome-like organelles were frequently observed in HPS2-AOs (Figure S3G), consistent with a previous report on Pearl mice (Zhen et al., 1999). We therefore concluded that altered distribution and enlargement of LBs with abnormally formed organelles were the phenotype of HPS2-AOs.

### Impaired Surfactant Secretion from HPS2-AO-Derived Cells

As decreased secretion of pulmonary surfactant was reported in HPS1 and HPS2 double-mutant mice (Pale Ear/Pearl) (Guttentag et al., 2005), we evaluated the pulmonary surfactant secretion of HPS2-AOs and cHPS2-AOs. EpCAM$^{+}$ cells of both AOs were reseeded on coverglass chambers, and live cell imaging was performed after staining with LT and FM1-43, a fluorescent probe that labels free lipids and which has been used to evaluate the exocytosis of LBs from primary AT2 cells (Haller et al., 1998) (Figure 4A). After stimulation with a secretagog cocktail, the fluorescence intensity of FM1-43 was increased in cHPS2-AO epithelial cells but not in HPS2-AO epithelial cells (Figures 4B and 4C; Video S1). Consistently, z-stack images suggested that more FM1-43 stained vesicles were secreted from cHPS2-AO cells than from HPS2-AO cells (Figure 4D). Furthermore, the concentration of phosphatidylcholine in culture supernatant was significantly reduced in HPS2-AO-derived cells (Figure 4E). These findings suggested that HPS2-AO cells were less prone to secrete the surfactant, consistent with the findings that Pearl mice showed decreased secretion of kidney lysosomal enzymes (Novak and Swank, 1979). In addition, there was no significant change in the levels of FM1-43 augmentation between the enlarged and normal LBs in HPS2-AOs (Figures 4B, S3H, and S3I), suggesting that impaired LB secretion in HPS2-AO cells was caused by dysfunction of the AP-3 complex rather than the size of the LBs.

## DISCUSSION

We established the HPS2-iPSCs from patient fibroblasts and their gene-corrected cHPS2-iPSCs. Although the mutation in exon 15 persisted in cHPS2-iPSCs, we consider the results obtained from the experiments using cHPS2-iPSCs

(C) qRT-PCR of fibrosis-related markers in AOs (P0) (mean ± SEM, n = 5 independent experiments). The Mann-Whitney test was used. n.s., not significant.
(D) Confocal IF staining of AOs (P0) derived from HPS2-iPSCs and cHPS2-iPSCs. Scale bars, 10 μm.
(E) qRT-PCR of *SFTPC* and *SLC34A2* in NaPi2b$^{high}$ and NaPi2b$^{low}$ cells sorted from AOs (P0) (mean ± SEM, n = 3 independent experiments).
(F) Schematic overview of the subculture of NaPi2b$^{high}$ cells in AOs. NaPi2b$^{high}$ cells were passaged every 2 weeks.
(G) Confocal IF staining of AOs (P2) subcultured using anti-NaPi2b antibodies. Scale bars, 10 μm.
(H) qRT-PCR of AT2 markers in subcultured AOs (P0–P2) (mean ± SEM, n = 3 independent experiments).
See also Figures S2 and S3.

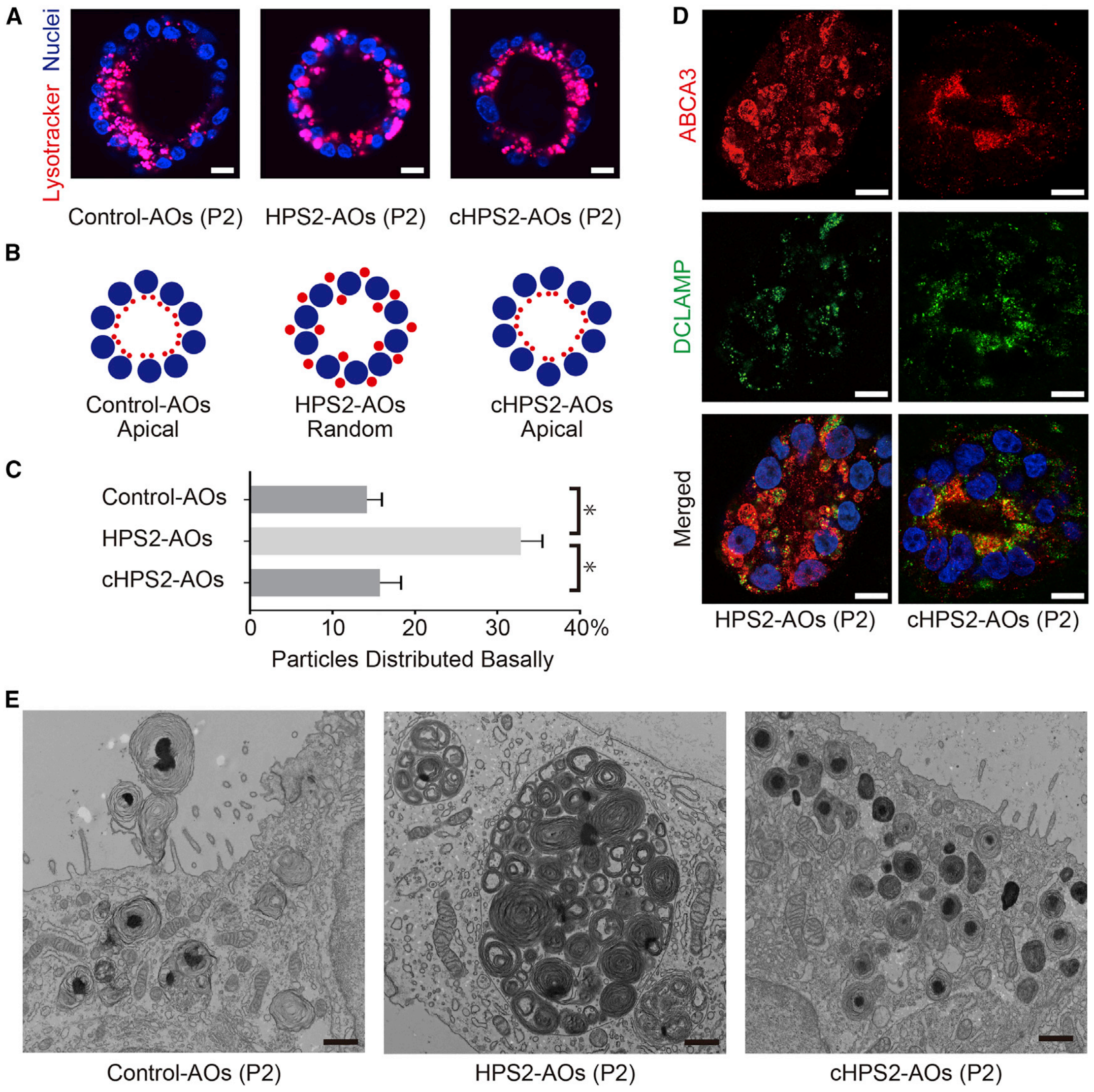


**Figure 3. Morphological Features of HPS2-AOs and cHPS2-AOs**

(A) Live cell imaging of AOs (P2) derived from control iPSCs, HPS2-iPSCs, and cHPS2-iPSCs, respectively. Scale bars, 10 μm.

(B) Schematic illustration of the pattern of $LT^+$ organelle distribution in AOs.

(C) Quantitative analysis of $LT^+$ organelles distributed basally in the spheroids (mean ± SEM, n = 22–32 from three independent experiments). Kruskal-Wallis with Dunn's multiple comparisons test was used. $*p < 0.05$.

(D) Confocal IF staining of ABCA3 and DCLAMP in HPS2-AOs and cHPS2-AOs (P2). Scale bars, 10 μm.

(E) Electron micrographs of AOs (P2) from each cell line. Scale bars, 1 μm.

See also Figure S3.

to be reliable for three reasons. First, the increased CD63 in HPS2-iPSCs was restored in cHPS2-iPSCs, suggesting that AP-3 complex works sufficiently, even in the presence of the exon 15 mutation. Second, HPS2 is an autosomal recessive disease and it was reported that the mother of the donor, who had the nonsense mutation in exon 15 but not in exon 18, had no symptoms of HPS2 (Huizing et al., 2002). Finally, the amount of *AP3B1* mRNA is very low in HPS2-iPSCs due to NMD, and the aberrant protein production is negligibly low. For these reasons, we consider cHPS2-iPSCs to be an appropriate control for HPS2-iPSCs.

The mechanism underlying the formation of giant LBs in HPS is unknown. Since there was a report suggesting that enlarged lysosomes have impaired motility, even in wild-type cells (Perou and Kaplan, 1993), we investigated whether the size of LBs affected the FM1-43 augmentation

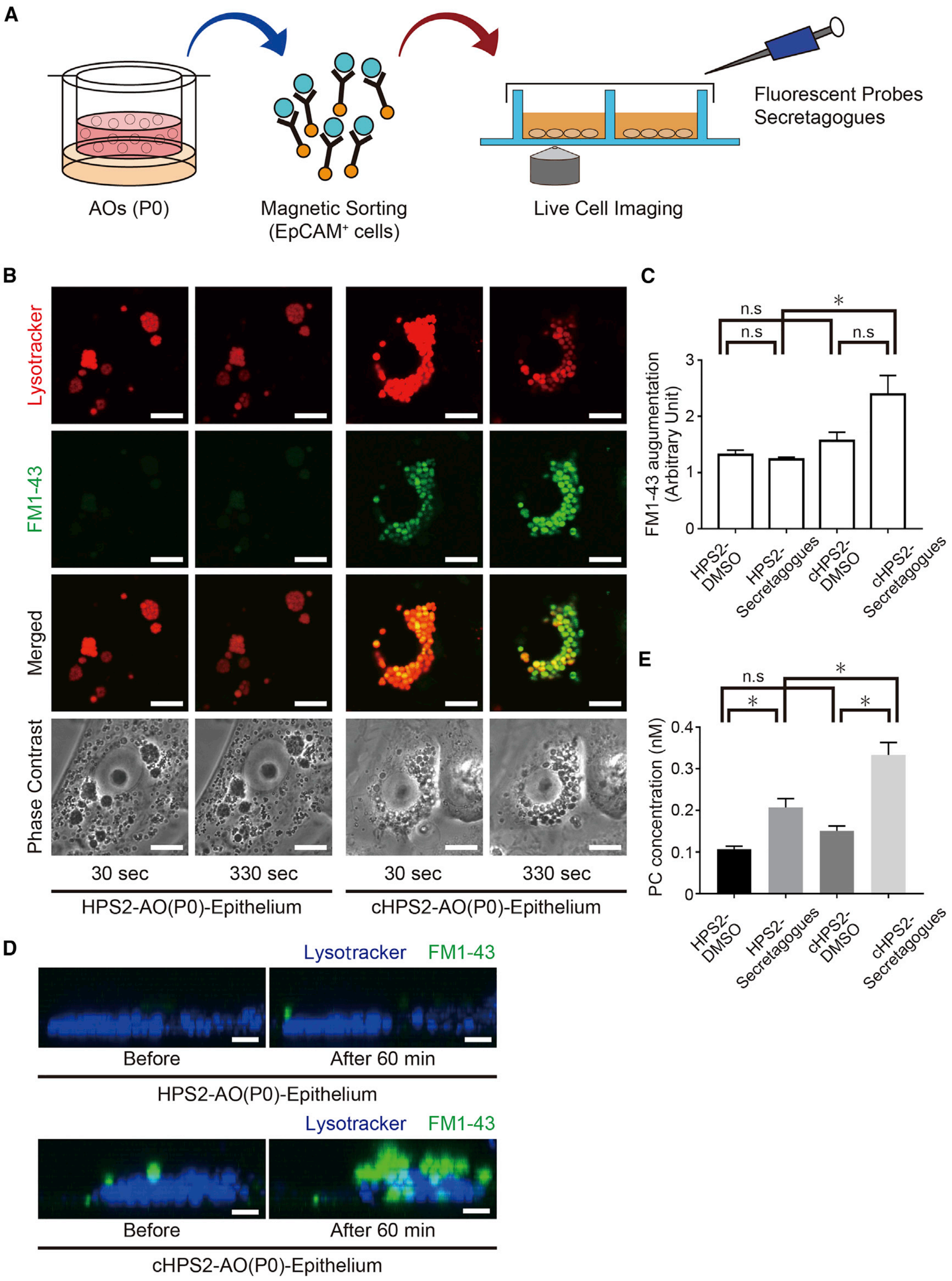


**Figure 4. LB Secretion Assay for AT2 Cells Derived from HPS2-iPSCs and cHPS2-iPSCs**
(A) Schematic overview of the LB secretion assay.
(B) Confocal images of HPS2-AO(P0) and cHPS2-AO(P0) epithelial cells at 30 and 330 s after stimulation with secretagogs. Phase-contrast images show the location of LBs and nuclei. Scale bars, 10 μm.

*(legend continued on next page)*

levels in the HPS2-AO epithelium. Our results suggested that the impaired LB secretion in HPS2-AO cells was caused by the dysfunction of AP-3 complex rather than the size of the LBs, which was consistent with the idea that AP-3 is required for the sorting of proteins involved in the movement of LROs along microtubules (Clark et al., 2003). We considered that the enlargement of the LBs in HPS2-AOs is a result of impaired surfactant secretion rather than its cause. In addition, the abnormal distribution of LBs seen in HPS2-AOs was analogous to the previous report on abnormalities in cytotoxic T lymphocytes (CTLs) in HPS2 patients, in which lytic granules, the LROs of CTLs, failed to move to the secretory surface of the cells (Clark et al., 2003).

Pulmonary surfactant plays critical roles in the maintenance of the alveolar environment and its deficiency causes respiratory distress syndrome (RDS) in newborns. In addition, mutations in genes associated with LBs, such as *ABCA3* and *SFTPB*, are typically lethal in neonates because of defective surfactant metabolism (Whitsett et al., 2015). In this study, the stimulated secretion of pulmonary surfactant was decreased in HPS2-AOs, but it was reported that HPS2 patients do not present RDS and that Pearl mice do not develop RDS or PF spontaneously in their lifetimes (Young et al., 2007). Our finding that basal secretion was preserved, not abrogated, in HPS2-AOs might explain why HPS2 patients and Pearl mice do not present RDS. Further studies are needed to investigate this discrepancy. It also remains to be elucidated whether decreased surfactant secretion may play some roles in the pathogenesis of PF in HPS2 patients, although decreased surfactant secretion has been reported in PF patients (Schmidt et al., 2002).

In this study, we validated three methods related to lung stem cell research. First, for the isolation NKX2-1$^{+}$ lung progenitor cells, CPM-based and CD47-based methods were compared (Gotoh et al., 2014; Hawkins et al., 2017). The CPM$^{high}$ population contained more NKX2-1$^{+}$ cells than the CD47$^{high}$CD26$^{low}$ population, although our induction protocol had originally been developed to fit CPM-based sorting. The second involved the isolation of AT2 cells from the adult human lung by the anti-NaPi2b antibody. Currently, HT2-280 is used for isolating AT2 cells from adult lung (Gonzalez et al., 2010). We demonstrated that the proportions of SFTPC$^{+}$ AT2 cells in the NaPi2b$^{high}$ and the HT2-280$^{high}$ populations were equivalent and that other AT2 cell markers were enriched in the NaPi2b$^{high}$ population. Finally, we demonstrated that the anti-NaPi2b antibody was also useful for passaging hPSC-derived AOs without establishing reporter cell lines. As we previously reported, the *SFTPC* expression in hPSC-AOs decreased without passaging, and it was necessary to isolate SFTPC$^{+}$ cells and reseed them into Matrigel with HFLFs in order to maintain the AT2 marker expression (Yamamoto et al., 2017). Although this is a limitation of the current hPSC-AO system, progress has been made in maintaining more AT2 markers in organoids in comparison with conventional two-dimensional culture. In this study, P2-AOs were used to analyze the distribution or morphological characteristics of LBs because P2-AOs contained more LBs than P0-AOs (Figure 3). On the other hand, to analyze the LB secretion (Figure 4), EpCAM$^{+}$ cells, not NaPi2b$^{high}$ cells, from P0-AOs were used to obtain sufficient cells.

It is necessary for disease modeling to recapitulate the functions of the cells responsible for the disease. While hPSC-derived lung organoids including both airway and alveolar lineages have been reported (Chen et al., 2017), there are few reports of organoids focusing on alveolar cells and their functions (Jacob et al., 2017; Yamamoto et al., 2017). To our knowledge, no reports have verified the secretion of pulmonary surfactant in both hPSC-derived AOs and in HPS patient-derived cells. Human iPSC-derived AT2 cells might overcome the limited availability and aid in discovering therapeutic agents via disease modeling in a dish.

## EXPERIMENTAL PROCEDURES

### Live Cell Imaging

After AOs or reseeded cells were stained with fluorescent probes, all of the samples were examined under an FV10i-LIV confocal microscope (Olympus) with a 60× objective under 5% $CO_2$ at 37°C. For the LB secretion assay, the medium was supplemented with a secretagog cocktail so that the final concentration of each component was 5 μM forskolin, 15 μM ATP, 150 nM ionomycin, and 150 nM phorbol 12-myristate 13-acetate. For further details, see Supplemental Experimental Procedures.

### Ethics

The use of H9 hESCs was approved by the Ministry of Education, Culture, Sports, Science and Technology (MEXT), Japan. The animal experiments were approved by the Animal Research

(C) Quantitative comparison of the FM1-43 augmentation levels (mean ± SEM, n = 7–10 from three independent experiments). Kruskal-Wallis with Dunn's multiple comparisons test was used. *p < 0.05; n.s., not significant.
(D) z-stack images of LT and FM1-43 staining before and after stimulation with secretagogs. Scale bars, 5 μm.
(E) Phosphatidylcholine (PC) concentration of the supernatant of secretagog-stimulated epithelial cells from AOs (mean ± SEM, n = 11 from 11 independent experiments). Kruskal-Wallis with Dunn's multiple comparisons test was used. *p < 0.05; n.s., not significant.
See also Figure S3 and Video S1.

Committee of Kyoto University. The use of human lung samples was approved by the Ethics Committee of Kyoto University Graduate School and Faculty of Medicine.

### Statistical Analyses

All error bars indicate the SEM. Quantified data represent the findings of three or more independent experiments. The statistical tests used are shown in each legend. All statistical analyses were performed using the Prism7 software program (GraphPad).

## SUPPLEMENTAL INFORMATION

Supplemental Information includes Supplemental Experimental Procedures, three figures, four tables, and one video and can be found with this article online at https://doi.org/10.1016/j.stemcr.2019.01.014.

## AUTHOR CONTRIBUTIONS

Y.K. and S.G. conceived and designed the study. Y.K., S.G., S.I., Y.Y., N.S., K.T., S.K., and T.N. performed the experiments. Y.K., S.G., I.A., and A.H. generated HPS2-iPSCs and cHPS2-iPSCs. Y.K., S.G., S.I., Y.Y., T.F.C.-Y., and H.D. contributed to AT2 cell isolation from human lung tissues. Y.K., S.G., S.I., and Y.Y. analyzed the data. Y.K. and S.G. wrote the manuscript through fruitful discussions with and supervision by H.M., I.I., M.H., M.M., and T.H.

## ACKNOWLEDGMENTS

We thank A. Nagahashi, K. Okita, K. Osafune, and S. Yamanaka (Center for iPS Cell Research and Application, Kyoto University) for iPSC-related materials and support, and T. Maruyama, S. Kanagaki, K. Moriguchi, T. Suezawa, M. Toyomoto, Y. Maeda, Y. Koyama, Y. Okuno and all the members of Medical Research Support Center, Kyoto University for technical assistance and consultation. We thank all the members of the Department of Thoracic Surgery, Kyoto University for providing surgical specimens of human adult lung, N. Inagaki and D. Tanaka (Department of Diabetes, Endocrinology and Nutrition, Kyoto University) for kindly providing anti-ABCA3 antibody, G. Ritter (Ludwig Institute for Cancer Research, New York City, USA) for kindly providing anti-NaPi2b antibody (MX35), and K. Okamoto-Furuta and H. Kohda (Division of Electron Microscopic Study, Center for Anatomical Studies, Kyoto University) for electron microscopy. This work was supported by JSPS KAKENHI (JP15K21114 and JP17H05084 to S.G. and JP15H02537 to M.M.), AMED (JP18bm0804007 to T.H. and JP18bm0704008 to S.G.), Takeda Science Foundation (S.G.), and in part by Kyorin Pharmaceutical.

Received: June 1, 2018
Revised: January 17, 2019
Accepted: January 17, 2019
Published: February 14, 2019

## REFERENCES

Barkauskas, C.E., Cronce, M.J., Rackley, C.R., Bowie, E.J., Keene, D.R., Stripp, B.R., Randell, S.H., Noble, P.W., and Hogan, B.L. (2013). Type 2 alveolar cells are stem cells in adult lung. J. Clin. Invest. *123*, 3025–3036.

Chen, Y.W., Huang, S.X., de Carvalho, A., Ho, S.H., Islam, M.N., Volpi, S., Notarangelo, L.D., Ciancanelli, M., Casanova, J.L., Bhattacharya, J., et al. (2017). A three-dimensional model of human lung development and disease from pluripotent stem cells. Nat. Cell Biol. *19*, 542–549.

Clark, R.H., Stinchcombe, J.C., Day, A., Blott, E., Booth, S., Bossi, G., Hamblin, T., Davies, E.G., and Griffiths, G.M. (2003). Adaptor protein 3-dependent microtubule-mediated movement of lytic granules to the immunological synapse. Nat. Immunol. *4*, 1111–1120.

Dell'Angelica, E.C., Shotelersuk, V., Aguilar, R.C., Gahl, W.A., and Bonifacino, J.S. (1999). Altered trafficking of lysosomal proteins in Hermansky-Pudlak syndrome due to mutations in the beta 3A subunit of the AP-3 adaptor. Mol. Cell *3*, 11–21.

El-Chemaly, S., and Young, L.R. (2016). Hermansky-Pudlak syndrome. Clin. Chest Med. *37*, 505–511.

Gonzalez, R.F., Allen, L., Gonzales, L., Ballard, P.L., and Dobbs, L.G. (2010). HTII-280, a biomarker specific to the apical plasma membrane of human lung alveolar type II cells. J. Histochem. Cytochem. *58*, 891–901.

Gotoh, S., Ito, I., Nagasaki, T., Yamamoto, Y., Konishi, S., Korogi, Y., Matsumoto, H., Muro, S., Hirai, T., Funato, M., et al. (2014). Generation of alveolar epithelial spheroids via isolated progenitor cells from human pluripotent stem cells. Stem Cell Reports *3*, 394–403.

Guttentag, S.H., Akhtar, A., Tao, J.Q., Atochina, E., Rusiniak, M.E., Swank, R.T., and Bates, S.R. (2005). Defective surfactant secretion in a mouse model of Hermansky-Pudlak syndrome. Am. J. Respir. Cell Mol. Biol. *33*, 14–21.

Haller, T., Ortmayr, J., Friedrich, F., Volkl, H., and Dietl, P. (1998). Dynamics of surfactant release in alveolar type II cells. Proc. Natl. Acad. Sci. U S A *95*, 1579–1584.

Hawkins, F., Kramer, P., Jacob, A., Driver, I., Thomas, D.C., McCauley, K.B., Skvir, N., Crane, A.M., Kurmann, A.A., Hollenberg, A.N., et al. (2017). Prospective isolation of NKX2-1-expressing human lung progenitors derived from pluripotent stem cells. J. Clin. Invest. *127*, 2277–2294.

Huizing, M., Scher, C.D., Strovel, E., Fitzpatrick, D.L., Hartnell, L.M., Anikster, Y., and Gahl, W.A. (2002). Nonsense mutations in ADTB3A cause complete deficiency of the beta3A subunit of adaptor complex-3 and severe Hermansky-Pudlak syndrome type 2. Pediatr. Res. *51*, 150–158.

Jacob, A., Morley, M., Hawkins, F., McCauley, K.B., Jean, J.C., Heins, H., Na, C.L., Weaver, T.E., Vedaie, M., Hurley, K., et al. (2017). Differentiation of human pluripotent stem cells into functional lung alveolar epithelial cells. Cell Stem Cell *21*, 472–488.e10.

Jessen, B., Bode, S.F., Ammann, S., Chakravorty, S., Davies, G., Diestelhorst, J., Frei-Jones, M., Gahl, W.A., Gochuico, B.R., Griese, M., et al. (2013). The risk of hemophagocytic lymphohistiocytosis in Hermansky-Pudlak syndrome type 2. Blood *121*, 2943–2951.

Konishi, S., Gotoh, S., Tateishi, K., Yamamoto, Y., Korogi, Y., Nagasaki, T., Matsumoto, H., Muro, S., Hirai, T., Ito, I., et al. (2016). Directed induction of functional multi-ciliated cells in proximal

airway epithelial spheroids from human pluripotent stem cells. Stem Cell Reports *6*, 18–25.

Kook, S., Qi, A., Wang, P., Meng, S., Gulleman, P., Young, L.R., and Guttentag, S.H. (2018). Gene-edited MLE-15 cells as a model for the Hermansky-Pudlak syndromes. Am. J. Respir. Cell Mol. Biol. *58*, 566–574.

Li, H.L., Fujimoto, N., Sasakawa, N., Shirai, S., Ohkame, T., Sakuma, T., Tanaka, M., Amano, N., Watanabe, A., Sakurai, H., et al. (2015). Precise correction of the dystrophin gene in duchenne muscular dystrophy patient induced pluripotent stem cells by TALEN and CRISPR-Cas9. Stem Cell Reports *4*, 143–154.

Lyerla, T.A., Rusiniak, M.E., Borchers, M., Jahreis, G., Tan, J., Ohtake, P., Novak, E.K., and Swank, R.T. (2003). Aberrant lung structure, composition, and function in a murine model of Hermansky-Pudlak syndrome. Am. J. Physiol. Lung Cell. Mol. Physiol. *285*, L643–L653.

Nakatani, Y., Nakamura, N., Sano, J., Inayama, Y., Kawano, N., Yamanaka, S., Miyagi, Y., Nagashima, Y., Ohbayashi, C., Mizushima, M., et al. (2000). Interstitial pneumonia in Hermansky-Pudlak syndrome: significance of florid foamy swelling/degeneration (giant lamellar body degeneration) of type-2 pneumocytes. Virchows Arch. *437*, 304–313.

Novak, E.K., and Swank, R.T. (1979). Lysosomal dysfunctions associated with mutations at mouse pigment genes. Genetics *92*, 189–204.

Perou, C.M., and Kaplan, J. (1993). Chediak-Higashi syndrome is not due to a defect in microtubule-based lysosomal mobility. J. Cell Sci. *106*, 99–107.

Rous, B.A., Reaves, B.J., Ihrke, G., Briggs, J.A., Gray, S.R., Stephens, D.J., Banting, G., and Luzio, J.P. (2002). Role of adaptor complex AP-3 in targeting wild-type and mutated CD63 to lysosomes. Mol. Biol. Cell *13*, 1071–1082.

Schmidt, R., Meier, U., Markart, P., Grimminger, F., Velcovsky, H.G., Morr, H., Seeger, W., and Gunther, A. (2002). Altered fatty acid composition of lung surfactant phospholipids in interstitial lung disease. Am. J. Physiol. Lung Cell. Mol. Physiol. *283*, L1079–L1085.

Whitsett, J.A., Wert, S.E., and Weaver, T.E. (2015). Diseases of pulmonary surfactant homeostasis. Annu. Rev. Pathol. *10*, 371–393.

Yamamoto, Y., Gotoh, S., Korogi, Y., Seki, M., Konishi, S., Ikeo, S., Sone, N., Nagasaki, T., Matsumoto, H., Muro, S., et al. (2017). Long-term expansion of alveolar stem cells derived from human iPS cells in organoids. Nat. Methods *14*, 1097–1106.

Yamano, G., Funahashi, H., Kawanami, O., Zhao, L.X., Ban, N., Uchida, Y., Morohoshi, T., Ogawa, J., Shioda, S., and Inagaki, N. (2001). ABCA3 is a lamellar body membrane protein in human lung alveolar type II cells. FEBS Lett. *508*, 221–225.

Yin, B.W., Kiyamova, R., Chua, R., Caballero, O.L., Gout, I., Gryshkova, V., Bhaskaran, N., Souchelnytskyi, S., Hellman, U., Filonenko, V., et al. (2008). Monoclonal antibody MX35 detects the membrane transporter NaPi2b (SLC34A2) in human carcinomas. Cancer Immun. *8*, 3.

Young, L.R., Pasula, R., Gulleman, P.M., Deutsch, G.H., and McCormack, F.X. (2007). Susceptibility of Hermansky-Pudlak mice to bleomycin-induced type II cell apoptosis and fibrosis. Am. J. Respir. Cell Mol. Biol. *37*, 67–74.

Zhen, L., Jiang, S., Feng, L., Bright, N.A., Peden, A.A., Seymour, A.B., Novak, E.K., Elliott, R., Gorin, M.B., Robinson, M.S., et al. (1999). Abnormal expression and subcellular distribution of subunit proteins of the AP-3 adaptor complex lead to platelet storage pool deficiency in the pearl mouse. Blood *94*, 146–155.

**Stem Cell Reports, Volume *12***

## Supplemental Information

# *In Vitro* Disease Modeling of Hermansky-Pudlak Syndrome Type 2 Using Human Induced Pluripotent Stem Cell-Derived Alveolar Organoids

**Yohei Korogi, Shimpei Gotoh, Satoshi Ikeo, Yuki Yamamoto, Naoyuki Sone, Koji Tamai, Satoshi Konishi, Tadao Nagasaki, Hisako Matsumoto, Isao Ito, Toyofumi F. Chen-Yoshikawa, Hiroshi Date, Masatoshi Hagiwara, Isao Asaka, Akitsu Hotta, Michiaki Mishima, and Toyohiro Hirai**

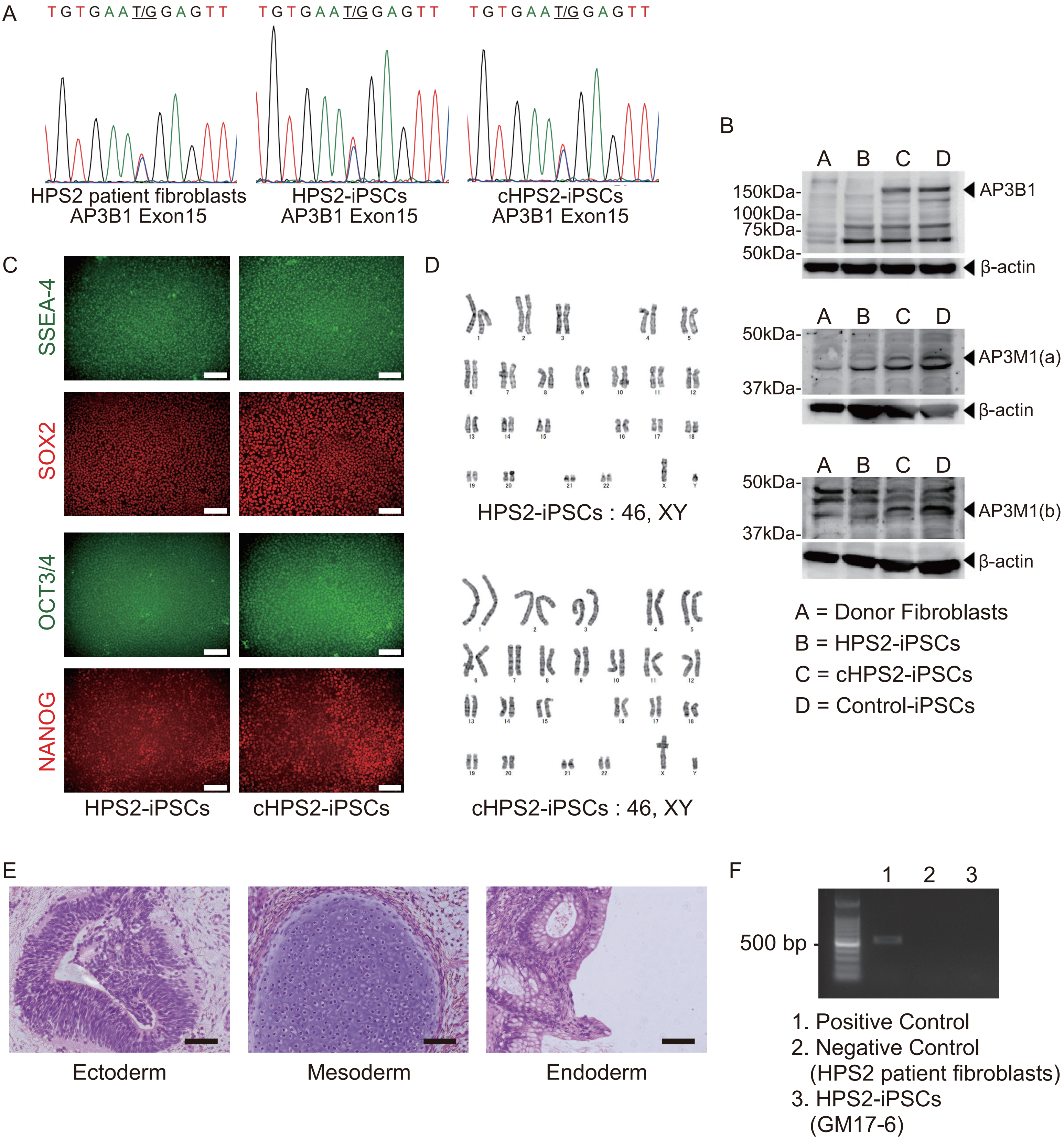


**Figure S1. Generation of HPS2-iPSCs and cHPS2-iPSCs, Related to Figure 1.**
(A) Sequence data of exon 15 in donor fibroblasts, HPS2-iPSCs and cHPS2-iPSCs. The mutation in exon 15 was not corrected in cHPS2-iPSCs. (B) Western blotting analyses of AP3B1 and AP3M1. AP3M1 was analyzed with two primary antibodies ((a) NBP1-76589 and (b) NBP2-27068). 201B7 iPSCs were used for control iPSCs. (C) IF staining of undifferentiated markers SSEA-4, SOX2, OCT3/4 and NANOG in HPS2-iPSCs and cHPS2-iPSCs. Scale bars, 100 μm. (D) The G-banding analysis of the karyotypes of HPS2-iPSCs and cHPS2-iPSCs. (E) Hematoxylin-eosin staining of teratoma derived from HPS2-iPSCs in immunodeficient mice. The three panels show ectoderm (neuronal cells), mesoderm (cartilage), and endoderm (gland) tissues. Scale bars, 100 μm. (F) The PCR to detect the integration of reprogramming vectors in genomic DNA. PCR products were not detected in HPS2-iPSCs.

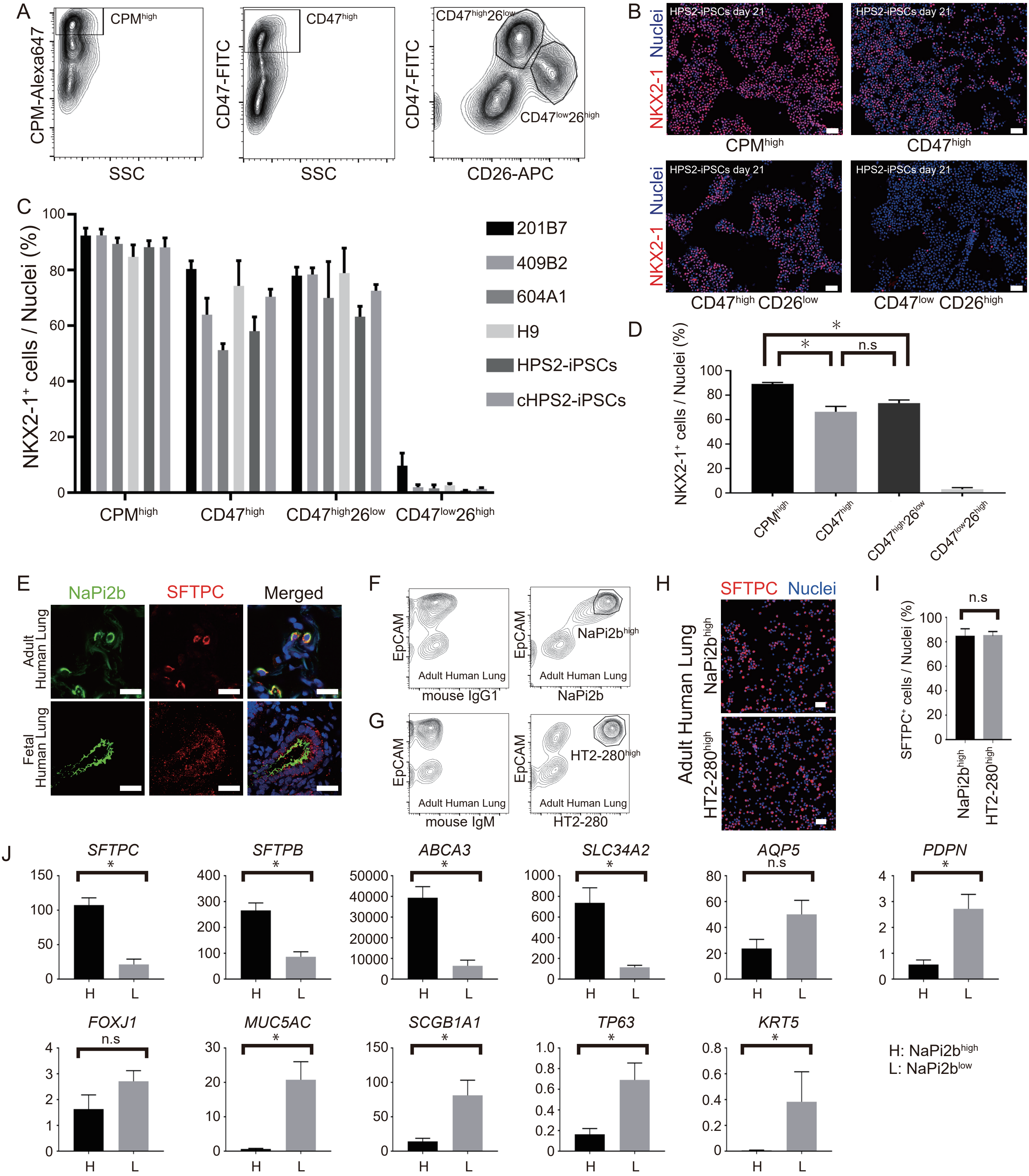


**Figure S2. Validation of Methods of Isolating NKX2-1⁺ Cells and Passaging of AT2 Cells Based on the NaPi2b Expression, Related to Figure 2.**

(A) Flow cytometric analyses of iPSC-derived lung progenitor cells on day 21. The gating of CPM$^{high}$, CD47$^{high}$, CD47$^{high}$CD26$^{low}$ and CD47$^{low}$CD26$^{high}$ are shown. (B) IF staining of cytospin samples of each cell population with anti-NKX2-1 antibody. Scale bars, 50μm. (C, D) The quantitative comparison of the proportion of NKX2-1$^{+}$ cells (mean ± S.E.M., n = 3 for (C) and n = 18 for (D), 3 independent experiments). A two-way ANOVA with Tukey’ s multiple comparisons test was used. *$p < 0.05$. n.s., not significant. (E) Confocal IF staining of adult and fetal human lung tissues with anti-NaPi2b and anti-SFTPC antibodies. Scale bars, 25 μm. (F, G) Flow cytometric analyses of adult human lung. CD45-negative cells are plotted and the gating of NaPi2b$^{high}$ and HT2-280$^{high}$ is shown. (H) IF staining of cytospin samples isolated from adult human lung. Scale bars, 50 μm. (I) The proportion of SFTPC$^{+}$ cells in NaPi2b$^{high}$ and HT2-280$^{high}$ cell population (mean ± S.E.M., n = 3 donors). The Mann-Whitney test was used. n.s., not significant. (J) The transcript levels of lineage-specific markers of the NaPi2b$^{high}$ and NaPi2b$^{low}$ population from adult human lung (mean ± S.E.M., n = 4 donors). The Mann-Whitney test was used. *$p < 0.05$. n.s., not significant.

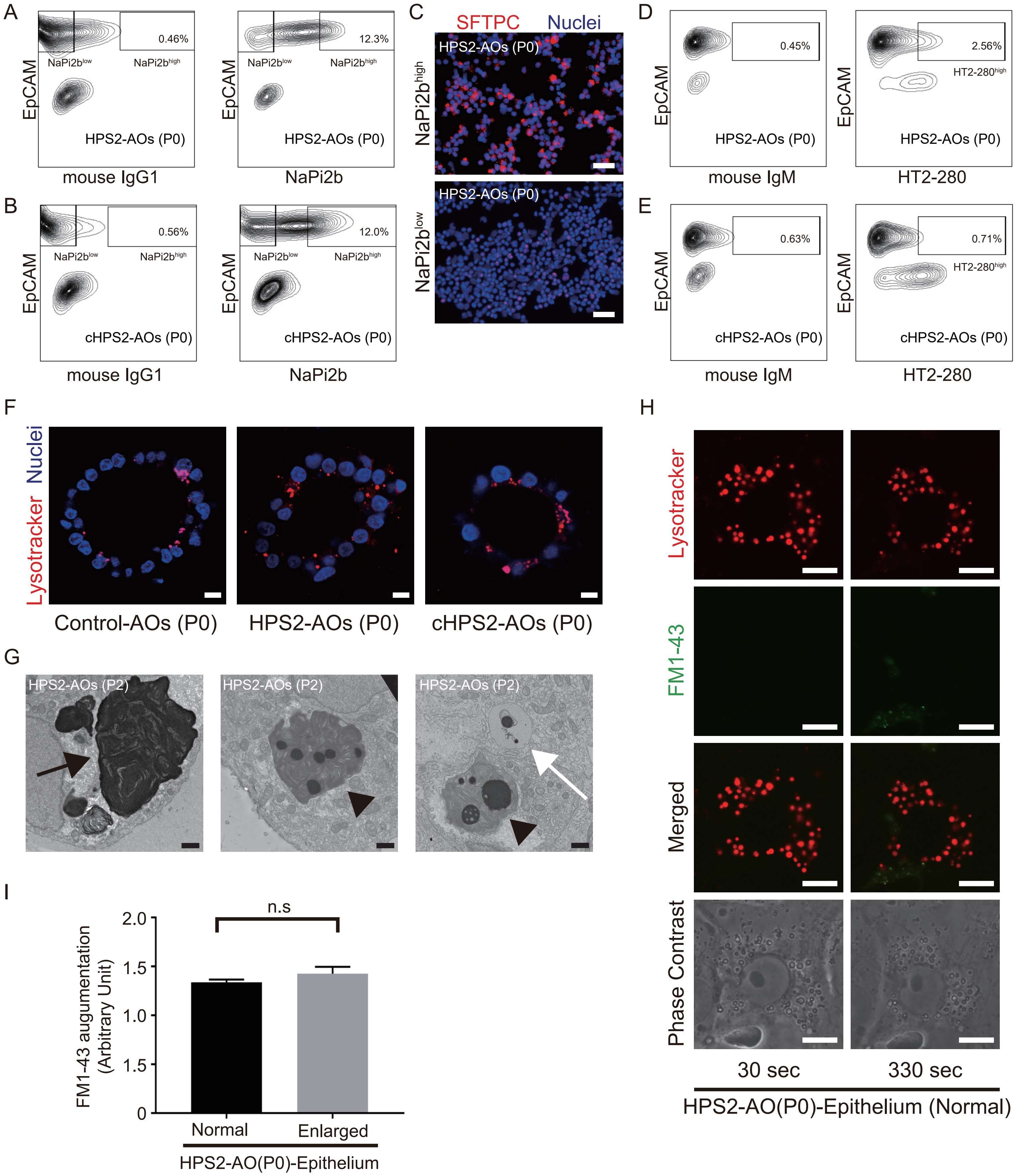

A
B
C
D
E
F
G
H
I
EpCAM
NaPi2b$^{low}$
NaPi2b$^{high}$
0.46%
12.3%
0.56%
12.0%
HPS2-AOs (P0)
cHPS2-AOs (P0)
mouse IgG1
NaPi2b
SFTPC
Nuclei
NaPi2b$^{high}$
NaPi2b$^{low}$
0.45%
2.56%
0.63%
0.71%
HT2-280$^{high}$
mouse IgM
HT2-280
Lysotracker
Control-AOs (P0)
HPS2-AOs (P2)
FM1-43
Merged
Phase Contrast
30 sec
330 sec
HPS2-AO(P0)-Epithelium (Normal)
n.s
FM1-43 augumentation (Arbitrary Unit)
2.0
1.5
1.0
1.5
0
Normal
Enlarged
HPS2-AO(P0)-Epithelium

**Table S1. Predicted off-target sites for CRISPR-sgRNA, Related to Figure 1.**

| chromosome | strand | start | end | sequence |
|---|---|---|---|---|
| chr3 | + | 85269952 | 85269972 | CGTTT--ACAGGCAAAATAATGG |
| chr4 | + | 84242401 | 84242422 | AGTTTAAAC-GTCAAAATAATGG |
| chr5 | + | 122400128 | 122400150 | GGTATAGACAGGCAAAACAATGG |
| chr6 | + | 30393163 | 30393185 | GGTTTATAA-AAGCAAAATAATGG |
| chr6 | + | 60699704 | 60699726 | GGATTAAATAGTCAAAATAATGG |
| chr6 | + | 101860608 | 101860628 | GGTCT-AACAGGCAAAATAA-GG |
| chr6 | + | 144457237 | 144457260 | GGTTTAAAGAGGCAAAATGAATGG |
| chr6 | + | 152250503 | 152250524 | GGTCTAAA-AAGCAAAATAATGG |
| chr6_GL000250v2_alt | + | 1722659 | 1722681 | GGTTTATAA-AAGCAAAATAATGG |
| chr6_GL000251v2_alt | + | 1872847 | 1872869 | GGTTTATAA-AAGCAAAATAATGG |
| chr6_GL000252v2_alt | + | 1649013 | 1649035 | GGTTTATAA-AAGCAAAATAATGG |
| chr6_GL000253v2_alt | + | 1703400 | 1703422 | GGTTTATAA-AAGCAAAATAATGG |
| chr6_GL000254v2_alt | + | 1737213 | 1737235 | GGTTTATAA-AAGCAAAATAATGG |
| chr6_GL000255v2_alt | + | 1648248 | 1648270 | GGTTTATAA-AAGCAAAATAATGG |
| chr6_GL000256v2_alt | + | 1691623 | 1691645 | GGTTTATAA-AAGCAAAATAATGG |
| chr7 | + | 3678866 | 3678887 | GGTATCAACAGGCAAAATAAT-G |
| chr7 | + | 8542826 | 8542847 | AGTTTACACAGGCAAAATAAT-G |
| chr9 | + | 84293388 | 84293408 | GGTTTAAACAGG--AAATTATGG |
| chr9 | + | 98542216 | 98542237 | GGTTTAAAAATGCAAAATAA-GG |
| chr9 | + | 99984208 | 99984229 | GGTTT-TATAGGCAAAATAATGG |
| chr9 | + | 112715180 | 112715200 | GGTTTAAA-AGACAAAA-AATGG |
| chr10 | + | 73652954 | 73652976 | GGTTCAAACA-GCAAAAATAATGG |
| chr10 | + | 102727399 | 102727422 | GGTTTAAACAGGCAGGAAAT-ATGG |
| chr11 | + | 119514483 | 119514505 | TGTTAAAAGAGGCAAAATAATGG |
| chr12 | + | 130173993 | 130174015 | TGTTAAAACAGACAAAATAATGG |
| chr16 | + | 83854456 | 83854477 | GGTTT-AATAGGCAAAAGAATGG |
| chr21 | + | 32070464 | 32070483 | GGTTTAAACA-GCAAAAT-AT-G |
| chr21 | + | 34916634 | 34916654 | GGTTTAGACAGGC-AAA-AATGG |
| chrX | + | 24881839 | 24881860 | GGTGAAAACAGGCAAAAT-ATGG |
| chrX | + | 96805068 | 96805091 | GGTTTAATATAGGCAAAATAATGG |
| chrY | + | 11502526 | 11502547 | GGATTAAACAGG-AAAAAAATGG |
| chr1 | - | 61652206 | 61652227 | GC-TTATTTTTCCTGTTTAAACC |
| chr1 | - | 152893492 | 152893511 | CCATTATTTTG-CT-TTT-AACC |
| chr2 | - | 15325113 | 15325137 | CCATTATTTTGCCTTTTTGTAAACC |
| chr2 | - | 124743782 | 124743803 | CCATTTTTTTTCCTG-TTAAACC |
| chr2 | - | 150131432 | 150131453 | CCATTTATTTT-CCTGTTTAAA-C |
| chr2 | - | 199174432 | 199174452 | CCATT-TTTTACCT-TTTAAACC |
| chr3 | - | 27411420 | 27411440 | CCATTATTTT-CCT-TTTAAAGC |

| | | | | |
|---|---|---|---|---|
| chr3 | - | 70108850 | 70108870 | CAATT-TTTTG-CTGTTTAAACC |
| chr4 | - | 3013664 | 3013684 | CCTTTATTTTG-CTGTTTAAA-C |
| chr4 | - | 20408383 | 20408405 | CCATTAATTTGTCTGTTTGAACC |
| chr5 | - | 92717234 | 92717254 | CCA-T-TTTTGCCTGTTTATACC |
| chr6 | - | 83994041 | 83994063 | CCTTTCTTTTTCCTGTTTAAACC |
| chr6 | - | 147566614 | 147566637 | CAATTATTTTGCCAGGTTTAAACC |
| chr6 | - | 163497336 | 163497357 | CAATTATTTTG-CTGTTTAAAAC |
| chr7 | - | 102818353 | 102818375 | ACATTATTTAACCTGTTTAAACC |
| chr7 | - | 121948701 | 121948722 | CCATTATTTTG-TTATTTAAACC |
| chr8 | - | 135459209 | 135459230 | CCAGT-TTTTGCCTGTTTCAACC |
| chr10 | - | 11548921 | 11548940 | CCATTA-TTT-CCT-TTTAAACC |
| chr10 | - | 21218346 | 21218368 | TCTTTAATTTGCCTGTTTAAACC |
| chr10 | - | 23130755 | 23130775 | CCATTATTTCGCCTG-TTAAA-C |
| chr12 | - | 89207482 | 89207504 | CCATTATTTTTCCTATTTAAAAC |
| chr13 | - | 45752101 | 45752123 | CCATTATTTTGCCTGTTTTGTCC |
| chr13 | - | 95785431 | 95785452 | TCATTATTATGCCT-TTTAAACC |
| chr17 | - | 5432517 | 5432538 | TAATTATTTT-CCTGTTTAAACC |
| chr17 | - | 60139533 | 60139553 | CCGTTATTTTGCCT-TTT-AACC |
| chrX | - | 14360143 | 14360164 | CCTTTCTTTTGCCTG-TTAAACC |
| chrX | - | 88375144 | 88375165 | TCTTTATTTTGCCT-TTTAAACC |

**Table S2. Antibodies used in the present study. Related to Figures 1-4 and S1-3.**

| Species | Antibodies | Company | Catalog No./ Clone | Dilution |
|---|---|---|---|---|
| For isolating NKX2-1$^{+}$ cells | | | | |
| mouse | Anti-CPM | Abcam | ab49278/1C2 | 1:200 |
| mouse | Anti-CPM | Wako | 014-27501 | 1:200 |
| donkey | Anti-mouse IgG- Alexa Fluor 647 | Thermo Fisher Scientific | A-31571 | 1:200 |
| mouse | Anti-CD47-FITC | BioLegend | 323106/CC2C6 | 1:20 |
| mouse | Anti-CD26-APC | BioLegend | 302710/BA5b | 1:20 |
| mouse | Mouse IgG1-kappa-FITC | BD Pharmingen | 555748/MOPC-21 | 1:5 |
| mouse | Mouse IgG2a-kappa-APC | BD Pharmingen | 550882/G155-178 | 1:20 |
| For CD63 analysis | | | | |
| mouse | Anti-CD63- Alexa Fluor 647 | BioLegend | 353016/H5C6 | 1:20 |
| mouse | Mouse IgG1 kappa- Alexa Fluor 647 | BioLegend | 400130/MOPC-21 | 1:20 |
| For passage of AOs by MX35 | | | | |
| mouse | Anti-NaPi2b | kindly provided by Dr. Gerd Ritter (MX35) | | 1:100 |
| mouse | Mouse IgG1 | Sigma-Aldrich | M5284/MOPC21 | 1:100 |
| goat | Anti-EpCAM | R&D systems | AF960 | 1:100 |
| donkey | Anti-mouse IgG Alexa Fluor 647 | Thermo Fisher Scientific | A-31571 | 1:200 |
| donkey | Anti-goat IgG Alexa Fluor 488 | Thermo Fisher Scientific | A-11055 | 1:200 |
| For isolation of adult human AT2 cells | | | | |
| mouse | Anti-EpCAM | BioLegend | 324202/9C4 | 1:100 |
| mouse | Mouse IgG2b kappa | Thermo Fisher Scientific | 14-4732-82/ eBMG2b | 1:100 |
| mouse | Anti-CD45-VioBlue | Miltenyi Biotec | 130-092-880/5B1 | 1:100 |
| mouse | Mouse IgG2a-VioBlue | Miltenyi Biotec | 130-098-898/ S43.10 | 1:100 |
| mouse | Anti-NaPi2b | kindly provided by Dr. Gerd Ritter (MX35) | | 1:100 |
| mouse | Mouse IgG1 | Sigma-Aldrich | M5284/MOPC21 | 1:100 |
| mouse | HT2-280 | Terrace Biotech | TB-27AHT2-280 | 1:100 |
| mouse | Mouse IgM | Sigma-Aldrich | M5909/MOPC104E | 1:100 |
| goat | Anti-mouse IgG2b Alexa Fluor 488 | Thermo Fisher Scientific | A-21141 | 1:200 |
| goat | Anti-mouse IgG1 Alexa Fluor 647 | Thermo Fisher Scientific | A-21240 | 1:200 |
| donkey | Anti-mouse IgM Alexa Fluor 647 | Jackson ImmunoResearch | 715-605-140 | 1:50 |
| FM1-43 augmentation assay | | | | |
| mouse | Anti-EpCAM | Santa Cruz Biotechnology | sc-66020/EBA-1 | 1:100 |
| rat | Anti-mouse IgG1-microbeads | Miltenyi Biotec | 130-047-101 | 1:5 |
| For immunofluorescence staining | | | | |
| goat | Anti-β3A subunit (AP3B1) | abcam | AB118584 | 1:100 |
| rabbit | Anti-SFTPC | Santa Cruz Biotechnology | FL-197 | 1:100 |
| mouse | Anti-NaPi2b | kindly provided by Dr. Gerd Ritter (MX35) | | 1:100 |
| rabbit | Anti-ABCA3 | kindly provided by Prof. Nobuya Inagaki | | 1:500 |
| mouse | Anti-DCLAMP | Beckman Coulter | IM3448/104.G4 | 1:100 |

| | | | | |
|---|---|---|---|---|
| rabbit | Anti-NKX2-1 | abcam | ab76013/EP1584Y | 1:500 |
| mouse | Anti-SSEA-4 | BioLegend | 330401/MC-813-70 | 1:100 |
| rabbit | Anti-SOX2 | Millipore | AB5603 | 1:500 |
| mouse | Anti-OCT3/4 | Santa Cruz Biotechnology | sc-5279/C-10 | 1:100 |
| rabbit | Anti-NANOG | ReproCELL | RCAB004P-F | 1:100 |
| donkey | Anti-rabbit IgG-Cy3 | Jackson ImmunoResearch | 711-165-152 | 1:500 |
| donkey | Anti-mouse IgG-Alexa Fluor 488 | Thermo Fisher Scientific | A-21202 | 1:500 |
| donkey | Anti-goat IgG-Alexa Fluor 488 | Thermo Fisher Scientific | A-11055 | 1:500 |
| For Western blotting | | | | |
| rabbit | Anti-β3A subunit (AP3B1) | Proteintech | 13384-1-AP | 1:500 |
| rabbit | Anti-μ subunit (AP3M1) | Novus Biologicals | NBP1-76589 | 1:1000 |
| rabbit | Anti-μ subunit (AP3M1) | Novus Biologicals | NBP2-27068 | 1:1000 |
| mouse | Anti-$\beta$-actin | Sigma-Aldrich | A2228/AC-74 | 1:5000 |
| pig | Anti-rabbit immunoglobulins-HRP | Dako | P0399 | 1:1000 |

**Table S3. Content of the mediums. Related to Figures 2 and 4.**

<table>
<tr><th></th><th>Anteriorization medium</th><th>Ventralization medium</th><th>CFKD preconditioning medium</th></tr>
<tr><td>Basal Medium</td><td colspan="3">DMEM / F12<br>Glutamax<br>B27 supplement (2 %)<br>L-ascorbic acid (0.05 mg/ml)<br>monothioglycerol (0.4 mM)<br>Penicillin / Streptomycin (50 U/ml)</td></tr>
<tr><td>Chemicals / Cytokines</td><td>Noggin (100 ng/ml)<br>SB431542 (10 μM)</td><td>ATRA (depends on cell lines)<br>BMP4 (20 ng/ml)<br>CHIR99021 (depends on cell lines)<br>[Concentrations of ATRA/CHIR99021]<br>201B7: 0.05 μM/3.0 μM<br>409B2: 0.1 μM/2.5 μM<br>604A1: 1.0 μM/2.5 μM<br>H9: 0.5 μM/3.5 μM<br>HPS2- and cHPS2-iPSCs: 1.0 μM/3.5 μM</td><td>CHIR99021 (3 μM)<br>FGF10 (10 ng/ml)<br>KGF (10 ng/ml)<br>DAPT (20 μM)</td></tr>
<tr><th></th><th colspan="2">Alveolarization medium</th><th>Replating medium</th></tr>
<tr><td>Basal Medium</td><td colspan="3">Ham’s F12<br>B27 supplement (1 %)<br>BSA (0.25 %)<br>HEPES (15 mM)<br>$CaCl_2$ (0.8 mM)<br>ITS premix (0.1 %)<br>Penicillin / Streptomycin (50 U/ml)</td></tr>
<tr><td>Chemicals / Cytokines</td><td colspan="2">Dexamethasone (50 nM)<br>IBMX (100 μM)<br>KGF (10 ng/ml)<br>8-Br-cAMP (100 μM)</td><td>Dexamethasone (50 nM)<br>IBMX (100 μM)<br>KGF (10 ng/ml)<br>CHIR99021 (3 μM)<br>SB431542 (10 μM)</td></tr>
</table>

**Table S4. Primers used in the present study. Related to Figures 1-2 and S2.**

| Gene | Forward | Reverse | Size |
|---|---|---|---|
| *ABCA3* | TCTCCTTCAGCTTCATGGTCAG | TGGCTCAGAGTCATCCAGTTG | 136 |
| *ACTA2* | GGGTGACGAAGCACAGAGCA | CTTCAGGGGCAACACGAAGC | 137 |
| *AGER* | GCCACTGGTGCTGAAGTGTA | TGGTCTCCTTTCCATTCCTG | 191 |
| *AP3B1* | GAAGAATGCAGCCCATGCAA | TGCCAGCTACCAATGTGCTT | 116 |
| *AQP5* | CTGTCCATTGGCCTGTCTGTC | GGCTCATACGTGCCTTTGATG | 248 |
| *FOXJ1* | CCTGTCGGCCATCTACAAGT | AGACAGGTTGTGGCGGATT | 94 |
| *KRT5* | GAGCTGAGAAACATGCAGGA | TCTCAGCAGTGGTACGCTTG | 82 |
| *MUC5AC* | CATCTGCCAGCTGATTCTGA | AAGACGCAGCCCTCATAGAA | 129 |
| *PDPN* | TCCAGGAACCAGCGAAGAC | CGTGGACTGTGCTTTCTGA | 119 |
| *SCGB1A1* | CACCATGAAACTCGCTGTCAC | AGTTCCATGGCAGCCTCATAAC | 147 |
| *SFTPB* | GAGCCGATGACCTATGCCAAG | AGCAGCTTCAAGGGGAGGA | 133 |
| *SFTPC* | GCAAAGAGGTCCTGATGGAG | TGTTTCTGGCTCATGTGGAG | 178 |
| *SLC34A2* | TCGCCACTGTCATCAAGAAG | CTCTGTACGATGAAGGTCATGC | 112 |
| *SNAIL* | AATCCAGAGTTTACCTTCCAGCA | TCCCAGATGAGCATTGGCAG | 110 |
| *TGFB1* | GGAAATTGAGGGCTTTCGCC | CCGGTAGTGAACCCGTTGAT | 90 |
| *TP63* | ACTGCCAAATTGCAAAGACA | TGACTAGGAGGGGCAATCTG | 184 |
| *TWIST* | TCTCGGTCTGGAGGATGGAG | AATGACATCTAGGTCTCCGGC | 100 |
| *β-ACTIN* | CAATGTGGCCGAGGACTTTG | CATTCTCCTTAGAGAGAAGTGG | 126 |

## SUPPLEMENTAL EXPERIMENTAL PROCEDURES

### Generation of human iPSCs generation and maintenance of human PSCs

For the generation of HPS2-iPSCs, GM17890 HPS2 fibroblasts (NIGMS Human Genetic Cell Repository, Coriell Institute for Medical Research) were reprogrammed to iPSCs as previously reported (Okita et al., 2011). Human cDNAs of reprogramming factors were transduced into patient fibroblasts with episomal vectors (SOX2, KLF4, OCT3/4, L-MYC, LIN28, short hairpin RNA for p53). Several days after transduction, fibroblasts were harvested and replated on mitomycin C-treated STO feeder layer (DS Pharma Biomedical). The next day, the medium was changed to primate ES cell medium (Reprocell) supplemented with human FGF-2 (4 ng/ml) (Wako or DS Pharma Biomedical). The medium was then changed every other day. Thirty days after transduction, the iPSC colonies were picked up.

For the teratoma formation assay, undifferentiated HPS-iPSCs were harvested and re-suspended in DMEM/F12 supplemented with 10 μM of Y-27632 (LC laboratories). Approximately 5 x $10^5$ cells were injected into a testis of 8-week-old SCID mice (Clea Japan, Inc.). At 10-12 weeks after transplantation, the mice were sacrificed, and tumors were harvested and fixed with Mildform 10N (Wako) and embedded in paraffin. The sections were stained with hematoxylin and eosin.

For the maintenance of H9 human embryonic stem cells (hESCs), the cells were cultured on mitomycin C-treated STO feeder cells in Primate ES medium supplemented with 4 ng/ml human FGF-2 and 50 U/ml penicillin–streptomycin (Life Technologies). HPS2-iPSCs (GM17-6), cHPS2-iPSCs (res69-5) and control iPSCs (201B7, 409B2 and 604A1) were cultured in Essential 8 medium (Gibco) without feeder cells and used for differentiation studies within 20 passages.

### Gene correction of HPS2-iPSCs

The donor template was designed to have normal exon 18. Since AP3B1 is expressed ubiquitously, the splicing acceptor was placed upstream of the neomycin resistant gene in the donor template. HPS2-iPSCs were pretreated with Y-27632 at 10 μM for at least 1 h before electroporation. The cells were washed with PBS and treated with CTK solution for 1–3 min at room temperature (RT) to remove feeders and then washed with PBS twice. Next, the iPSCs were treated with accutase for 10 min at 37°C, dissociated into single cells by pipetting, and neutralized with culture medium. We electroporated 10 μg of Cas9 and sgRNA expression vectors (5 μg for Cas9 and 5 μg for sgRNA) and 5 μg of donor plasmid into 1 × $10^6$ cells using a NEPA 21 electroporator (poring pulse: pulse voltage, 125 V; pulse width, 5 ms; pulse number, 2; Nepagene). Cells were plated onto one well of a six-well plate with feeders in the presence of 10 μM Y-27632 for 1–2 days. G418 (Gibco) selection was applied after iPSC colonies were recovered (4–5 days after transfection). The resulting neomycin-resistant colonies were dissociated into single cells and plated at 200, 500 and 1500 cells per 10-cm dish with feeders. Each subclone was screened by genomic PCR and sequencing. After establishing the knockin clones, we electroporated the cells with 10 μg of the Cre expression vector using NEPA 21. Clone isolation was carried out and excision of the neomycin-selection cassette was confirmed by genomic PCR and sequencing.

Out of 132 isolated clones, 94 morphologically normal clones were screened for knock-in; 36 clones (27%) had the donor template at the target locus. After Cre excision, we chose a res69-5 clone for the subsequent experiments. For res69-5 clone, a total of 58 predicted off-target sites with up to 3 bp mismatches allowed (Table S1) (GGGenome; https://gggenome.dbcls.jp/ja/) were examined by sanger sequencing, resulting in no abnormal deletions or insertions being detected at these sites in the res69-5 cHPS2-iPSC clone.

### Western blotting

Thirty micrograms of protein in lysis buffer was loaded in each well of 10% polyacrylamide gels and electrophoresed at 100 V for 2 h. Proteins were transferred onto methanol-activated PVDF membrane (Amersham Hybond P PVDF 0.45) in transfer buffer

(20% methanol, 0% SDS) at 100 V for 1 h at 4°C. After being washed in TBS-T for 5 min, membranes were blocked with TBS-T containing 5% non-fat milk (Megmilk Snow Brand) for 1 h under agitation. Primary and secondary antibodies were diluted in TBS-T. Primary antibodies were incubated overnight and Secondary antibodies were incubated for 1 h under agitation, followed by washing with TBS-T for 5 min, three times. Signals were visualized by Clarity Western ECL Substrate (Bio-rad). The antibodies used are listed in Table S2.

**FACS analysis**

Single cell suspension was washed with FACS buffer (1% BSA /PBS supplemented with 10 μM Y-27632) and immunostained with primary antibodies at 4°C for 15 min. After being washed twice with FACS buffer, the cells were stained with secondary antibodies on demand at 4°C for 15 min. After being washed twice with FACS buffer, the cells were stained with propidium iodide (PI), and a FACS analysis was performed using a FACS Aria II or Aria III (BD Biosciences). The antibodies used are listed in Table S2.

**Induction and isolation of NKX2-1$^{+}$ lung progenitor cells.**

The differentiation of human PSCs was performed as previously described (Gotoh et al., 2014; Konishi et al., 2016; Yamamoto et al., 2017). In brief, undifferentiated human PSCs were differentiated into definitive endodermal cells on Geltrex (Gibco)-coated plates in RPMI medium (Nacalai Tesque) containing a saturated dose of Activin A (100 ng/ml) (Peprotech), 1 μM CHIR99021 (Axon Medchem), 2% B27 supplement (Life technologies) and 50 U/ml penicillin–streptomycin. The medium was replaced every two days. Y-27632 was supplemented on Day 0, and sodium butyrate were supplemented on Day 1, 2 and 4. During Day 6-10, the definitive endodermal cells were cultured in the anteriorization medium (Table S3), followed by switching on Day10 to the ventralization medium (Table S3) containing BMP4 (20 ng/ml) (HumanZyme) and adjusted doses of ATRA (Sigma-Aldrich)/CHIR99021 (Table S3). The optimized concentrations of ATRA/CHIR99021 for both HPS2-iPSCs (GM17-6) and cHPS2-iPSCs (res69-5) were 1.0 μM/3.5 μM. During Day 14-21, cells were cultured in CFKD preconditioning medium (Table S3). On Day 21, NKX2-1$^{+}$ lung progenitor cells were isolated using mouse anti-human CPM (Abcam or Wako) and anti-mouse IgG-Alexa647 (Invitrogen) for gating CPM$^{high}$ cells as previously reported (Yamamoto et al., 2017). To validate CD47/CD26-based isolation, mouse anti-human CD47-FITC (BioLegend) and mouse anti-human CD26-APC (BioLegend) were used for gating CD47$^{high}$ or CD47$^{high}$CD26$^{low}$ cells. Mouse IgG1-FITC (BD Pharmingen) and mouse IgG2a-APC (BD Pharmingen) were used for isotype controls. The antibodies used are listed in Table S2.

**AO formation**

A total of 1.0 × 10$^{4}$ CPM$^{high}$ cells and 5.0 × 10$^{5}$ human fetal lung fibroblasts were mixed in 100 μL of alveolarization medium (Table S3) supplemented with Y-27632 (10 μM) and 100 μL of Matrigel (Corning; 354230) and placed on a 12-well cell culture insert (Corning). The medium in the lower chamber was changed every two days. Human fetal lung fibroblasts (17.5 weeks of gestation; DV Biologics; PP002-F-1349, lot 121109VA) were cultured in DMEM (Nacalai Tesque) supplemented with 10% fetal bovine serum and used at passage number 9 or 10.

**AO passaging**

The duration of organoid culturing was 14±1 days. AO cells were dissociated with 0.1% Tripsin-EDTA at 37 °C for 15 minutes (Gotoh et al., 2014; Yamamoto et al., 2017), washed in FACS buffer twice, and immunostained with mouse anti-NaPi2b (MX35; kindly provided by Dr. Gerd Ritter) (Yin et al, 2008) and goat anti-EpCAM (R&D Systems). Anti-mouse IgG-Alexa647 (Invitrogen) and anti-goat IgG-Alexa488 (Invitrogen) were used as secondary antibodies. NaPi2b$^{high}$ and NaPi2b$^{low}$ cell populations were then isolated by FACS Aria II or III (BD Biosciences). A total of 1.0 × 10$^{4}$ NaPi2b$^{high}$ cells were mixed with 5.0

× $10^5$ human fetal lung fibroblasts in Matrigel and placed on a culture insert as described in AO formation. The antibodies used are listed in Table S2.

**qRT-PCR**

Total RNA was extracted with a PureLink RNA mini kit (Invitrogen). cDNA was prepared from 80 ng of total RNA per sample with SuperScript III reverse transciptase (Invitrogen), amplified by Power SYBR Green PCR Master Mix (Applied Biosystems) and quantified by QuantStudio 3 (Applied Biosystems). The gene expression was normalized to the β-actin expression and compared with that in control RNA of human fetal lung at 17, 18 and 22 weeks of gestation (Agilent Technologies; #540177, lot 0006055802) and was presented as the relative gene expression. The primers used in the present study are described in Table S4.

**IF staining**

Two-dimensional culture or slide samples were fixed with 4% paraformaldehyde/PBS for 15 min and permeabilized with 0.2% Triton X-100/PBS for 15 min. After 30 min of blocking in 5% normal donkey serum/1% BSA/PBS, they were immunostained with primary and secondary antibodies for 30 min each, as previously described (Gotoh et al, 2014). In contrast, AOs were fixed with 4% paraformaldehyde/PBS for 20 min and incubated in 30% sucrose/PBS overnight. Organoids were then embedded in OCT compound (Sakura Finetek) and frozen in liquid nitrogen. The frozen organoids were sliced into 10-µm-thick sections on slides, permeabilized and blocked as described above. They were then immunostained with primary antibodies overnight and with secondary antibodies for 1 h. Hoechst-33342 (Dojindo) was added to the secondary antibody solution in order to label nuclei. The antibodies used in the present study are listed in Table S2. For the quantitative analysis of IF imaging, images were taken by a BZ-X710 (Keyence) and quantified by Hybrid Cell Count/BZ-H3C (Keyence) from 10-15 areas. A TCS SP8 (Leica Microsystems) was used for confocal imaging, and an IX81 (Olympus) was used for other conventional IF imaging.

**Isolation of adult human AT2 cells from lung tissues**

Human AT2 cells were isolated as previously described, with some modifications (Fujino et al., 2012; Yamamoto et al., 2017). Lung tissues were obtained from nontumorous regions of surgically resected specimens from patients with lung tumors. Samples from 3 donors (77-year-old male, 64-year-old female, 62-year-old female) were used for the cytospin analysis and 4 donors (58-year-old female, 76-year-old male, 72-year-old female, 68-year-old male) were used for the qRT-PCR. Lung tissues were mechanically minced and dissociated with Dispase II (Wako), collagenase/Dispase (Sigma-Aldrich) and DNase I (Sigma-Aldrich) for 60 min at 37°C. After being filtered through a 100-µm mesh, the cells were treated with ACK lysing buffer (Gibco). For sorting with MX35 (anti-NaPi2b), cells were immunostained with primary antibodies of MX35, anti-human EpCAM and anti-human CD45-VioBlue and secondary antibodies of anti-mouse IgG2b-Alexa488 (Invitrogen) and anti-mouse IgG1-Alexa647 (Invitrogen). For sorting with HT2-280, HT2-280 (Terrace Biotec) and anti-mouse IgM-Alexa647 (Jackson ImmunoResearch) were used instead of MX35 and anti-mouse IgG1-Alexa 647, respectively. The antibodies and corresponding isotype controls used in the present study are listed in Table S2.

**Electron microscopy**

Small pieces of AOs were incubated in fixative solution consisting of 2.5% glutaraldehyde, 2% paraformaldehyde, 2% osmium tetroxide, 0.1% picric acid, 4% sucrose, and 0.1M phosphate buffer (pH 7.4) on ice for 2 h. *En bloc* staining was performed in 1% uranyl acetate at RT for 1 h, dehydrated, and embedded in Epon 812, as described previously (Gotoh et al., 2014). Ultrathin sections were stained with uranyl acetate and lead citrate and analyzed by transmission electron microscopy (Hitachi; H-7650).

**Live cell imaging**

AOs were stained with 10 µg/ml Hoechst 33342 and 100 nM Lysotracker Red DND-99 (Thermo Fisher Scientific; L7528) for 60 and 30 min, respectively. AOs were washed with PBS twice, placed on 35 mm glass-bottom dishes (Grainer) and examined under a FV10i-LIV confocal microscope (Olympus) under 5% $CO_2$ at 37°C. The apicobasal distribution of the Lysotracker-stained vesicles was quantified using the Image J software program (National Institutes of Health).

For the LB secretion assay, AOs were dissociated with 0.1% Tripsin-EDTA at 37°C for 15 min and stained with anti-EpCAM (Santa Cruz Biotechnology) followed by staining with anti-mouse IgG1-microbeads (Miltenyi Biotec), followed by separation using a LS column (Miltenyi Biotec). To obtain a sufficient number of cells, $EpCAM^+$ cells were used for the experiment. A total of $3.0 \times 10^5$ $EpCAM^+$ cells were reseeded on Geltrex-coated 8-well coverglass chambers (Thermo Fisher Scientific) in replating medium (Table S3) supplemented with 10 µM of Y-27632. At 24 ± 12 h after reseeding, cells were stained with Lysotracker Red DND-99 (100 nM) for 30 min, washed with PBS and given fresh replating medium. Before live cell imaging, the cells were incubated in 0.5 µM of FM1-43 (Thermo Fisher; T3163). Time-lapse imaging was then performed after the supplementation of the secretagogue cocktail prepared as stock solutions of forskolin (Dekkers et al., 2013), ATP (Haller et al., 1998), ionomycin (Neuland et al., 2014) and phorbol 12-myristate 13-acetate (PMA) (Neuland et al., 2014). The LB content secretion was quantified by calculating the FM1-43 augmentation level as "the change in the FM1-43 integral density divided by the change in the Lysotracker integral density". For the comparison of enlarged and normal LBs in HPS2-AO, LBs of>4 µm in diameter were analyzed as "enlarged"; LBs of ≤4 µm in diameter were analyzed as "normal". Image J was used to analyze images. Z-stack images were obtained before and 60 min after stimulation with the secretagogue cocktail. Lysotracker Deep Red (Thermo Fisher Scientific; L12492) (100 nM) was used for Z-stack images.

**PC assay**

A total of $3.0 \times 10^5$ $EpCAM^+$ cells were reseeded on a 96-well flat bottom plate in replating medium (Table S3) supplemented with 10 µM of Y-27632. At 24 ± 12 h after reseeding, the medium was removed and replaced with 200 µl of HBSS containing secretagogue cocktail or DMSO. After 3 hours, culture supernatant was collected. A phosphatidylcholine assay kit (Sigma-Aldrich; MAK049) was used for the quantification.

**SUPPLEMENTAL REFERENCES**

Dekkers, J.F., Wiegerinck, C.L., de Jonge, H.R., Bronsveld, I., Janssens, H.M., de Winter-de Groot, K.M., Brandsma, A.M., de Jong, N.W., Bijvelds, M.J., Scholte, B.J., et al. (2013). A functional CFTR assay using primary cystic fibrosis intestinal organoids. Nat Med 19, 939-945.

Fujino, N., Kubo, H., Ota, C., Suzuki, T., Suzuki, S., Yamada, M., Takahashi, T., He, M., Suzuki, T., Kondo, T., et al. (2012). A novel method for isolating individual cellular components from the adult human distal lung. Am J Respir Cell Mol Biol 46, 422-430.

Neuland, K., Sharma, N., and Frick, M. (2014). Synaptotagmin-7 links fusion-activated $Ca^{2+}$ entry and fusion pore dilation. J Cell Sci 127, 5218-5227.

Okita, K., Matsumura, Y., Sato, Y., Okada, A., Morizane, A., Okamoto, S., Hong, H., Nakagawa, M., Tanabe, K., Tezuka, K., et al. (2011). A more efficient method to generate integration-free human iPS cells. Nat Methods 8, 409-412.
